# Unveiling the role of regulatory T cells in the tumor microenvironment of pancreatic cancer through single-cell transcriptomics and *in vitro* experiments

**DOI:** 10.3389/fimmu.2023.1242909

**Published:** 2023-09-11

**Authors:** Wei Xu, Wenjia Zhang, Dongxu Zhao, Qi Wang, Man Zhang, Qiang Li, Wenxin Zhu, Chunfang Xu

**Affiliations:** ^1^ Department of Gastroenterology, The First Affiliated Hospital of Soochow University, Suzhou, Jiangsu, China; ^2^ Shanghai Clinical College, Anhui Medical University, Shanghai, China; ^3^ Department of Respiratory Medicine, Shanghai Tenth People’s Hospital, Tongji University School of Medicine, Shanghai, China; ^4^ Department of Interventional Radiology, The First Affiliated Hospital of Soochow University, Suzhou, Jiangsu, China; ^5^ Department of Gastroenterology, Affiliated Hospital of Jiangsu University, Jiangsu University, Zhenjiang, China; ^6^ Department of Emergency Medicine, The Affiliated Hospital of Xuzhou Medical University, Xuzhou, Jiangsu, China; ^7^ The Laboratory of Emergency Medicine, School of the Secondary Clinical Medicine, Xuzhou Medical University, Xuzhou, China; ^8^ Department of General, Visceral, and Transplant Surgery, Ludwig-Maximilians-University Munich, Munich, Germany; ^9^ Department of Gastroenterology, Kunshan Third People’s Hospital, Suzhou, Jiangsu, China

**Keywords:** pancreatic cancer, single cell sequencing, tumor microenvironment, regulatory T Cell, WGCNA

## Abstract

**Background:**

In order to investigate the impact of Treg cell infiltration on the immune response against pancreatic cancer within the tumor microenvironment (TME), and identify crucial mRNA markers associated with Treg cells in pancreatic cancer, our study aims to delve into the role of Treg cells in the anti-tumor immune response of pancreatic cancer.

**Methods:**

The ordinary transcriptome data for this study was sourced from the GEO and TCGA databases. It was analyzed using single-cell sequencing analysis and machine learning. To assess the infiltration level of Treg cells in pancreatic cancer tissues, we employed the CIBERSORT method. The identification of genes most closely associated with Treg cells was accomplished through the implementation of weighted gene co-expression network analysis (WGCNA). Our analysis of single-cell sequencing data involved various quality control methods, followed by annotation and advanced analyses such as cell trajectory analysis and cell communication analysis to elucidate the role of Treg cells within the pancreatic cancer microenvironment. Additionally, we categorized the Treg cells into two subsets: Treg1 associated with favorable prognosis, and Treg2 associated with poor prognosis, based on the enrichment scores of the key genes. Employing the hdWGCNA method, we analyzed these two subsets to identify the critical signaling pathways governing their mutual transformation. Finally, we conducted PCR and immunofluorescence staining *in vitro* to validate the identified key genes.

**Results:**

Based on the results of immune infiltration analysis, we observed significant infiltration of Treg cells in the pancreatic cancer microenvironment. Subsequently, utilizing the WGCNA and machine learning algorithms, we ultimately identified four Treg cell-related genes (TRGs), among which four genes exhibited significant correlations with the occurrence and progression of pancreatic cancer. Among them, CASP4, TOB1, and CLEC2B were associated with poorer prognosis in pancreatic cancer patients, while FYN showed a correlation with better prognosis. Notably, significant differences were found in the HIF-1 signaling pathway between Treg1 and Treg2 cells identified by the four genes. These conclusions were further validated through *in vitro* experiments.

**Conclusion:**

Treg cells played a crucial role in the pancreatic cancer microenvironment, and their presence held a dual significance. Recognizing this characteristic was vital for understanding the limitations of Treg cell-targeted therapies. CASP4, FYN, TOB1, and CLEC2B exhibited close associations with infiltrating Treg cells in pancreatic cancer, suggesting their involvement in Treg cell functions. Further investigation was warranted to uncover the mechanisms underlying these associations. Notably, the HIF-1 signaling pathway emerged as a significant pathway contributing to the duality of Treg cells. Targeting this pathway could potentially revolutionize the existing treatment approaches for pancreatic cancer.

## 1 Introduction

Pancreatic cancer is a highly aggressive and malignant gastrointestinal tumor, primarily located in the head of the pancreas. Pancreatic ductal adenocarcinoma, originating from pancreatic ductal cells, accounts for the majority of cases (> 90%) (1). Several risk factors have been identified, including smoking (2), obesity (3), alcohol intake (4), diabetes (5), family history (6), and chronic pancreatitis (7), which contribute significantly to the development of pancreatic cancer. Unfortunately, the prognosis for pancreatic cancer is remarkably poor, with a mere 9% 5-year survival rate (8), This disease is responsible for nearly the same number of deaths (466,000) as the number of cases (496,000) (9). Furthermore, based on a study encompassing 28 European countries, pancreatic cancer is projected to surpass breast cancer and become the third leading cause of cancer-related deaths by 2025, as its incidence remains relatively stable compared to the declining incidence of breast cancer (10). Despite extensive research efforts, the comprehensive understanding of the numerous transcriptome changes occurring during pancreatic carcinogenesis remains elusive. Hence, the identification of key mRNAs involved in the pathogenesis of pancreatic cancer is crucial for unraveling its underlying mechanisms.

Many factors influence tumor progression, such as drug resistance and epigenetic changes in tumor cells (11, 12). The tumor microenvironment plays a pivotal role in tumor development (13–16). Cancer cells, through the secretion of various cytokines, chemokines, and other substances, can actively modulate their surroundings (17, 18). This functional alteration leads to the reprogramming of neighboring cells, enabling them to actively contribute to the tumor’s survival and growth (19, 20). Immune cells constitute a critical component of the TME and significantly influence this process (21, 22). Increasing evidence highlights the involvement of both innate immune cells (macrophages, neutrophils, dendritic cells, innate lymphocytes, myeloid-derived suppressor cells, and natural killer cells) and adaptive immune cells (T cells and B cells) in tumor progression within the TME (23–25). In 2006, the crucial role of regulatory T (Treg) cells, known for their negative regulation of autoimmunity, was discovered in the development of pancreatic cancer (26). Subsequent studies have further substantiated this finding, demonstrating that Treg cells are the most potent immunosuppressants known to dampen the activity of CD4+, CD8+, and NK cells (27). The immunosuppressive mechanisms mediated by Treg cells include the direct elimination of effector T cells and competition with effector T cells for antigen-presenting cells (28, 29). Therefore, understanding the secrets of the pancreatic cancer tumor microenvironment, particularly the role of Treg cells in pancreatic cancer, holds significant importance. It can shed light on the immunosuppressive mechanisms at play and help overcome the treatment challenges associated with pancreatic cancer (30). In the past, technical limitations greatly constrained research on microenvironment cells (31). However, the advent of single-cell sequencing technology has revolutionized the study of the patient tissues at the single-cell level, enabling a more comprehensive investigation of tumor microenvironments (32, 33).

However, investigations focusing on Treg cells using single-cell transcriptomics and traditional transcriptomics remain relatively scarce. Thus, our study aimed to utilize single-cell sequencing data to delve into the involvement of classical immunosuppressive Treg cells in the tumor microenvironment of pancreatic cancer, with the goal of identifying key characteristics of Treg cells. This research could offer novel insights into the mechanisms underlying pancreatic cancer. Notably, the novelty of this study lied in its pioneering exploration of the role of Treg cells in the pancreatic cancer microenvironment, employing state-of-the-art bioinformatics technology. Additionally, it presented a novel approach by combining single-cell sequencing with traditional transcriptomics analysis, thus providing fresh perspectives for ongoing oncology research.

## 2 Materials and methods

### 2.1 Data download and collation

We conducted a comprehensive search in the GEO database using the keyword ‘pancreatic cancer’ to identify relevant datasets containing gene expression levels in pancreatic cancer tissues. The inclusion criteria for the selected datasets were as follows: (1) the samples were derived from human pancreatic tissue, (2) the dataset comprised both tumor and normal tissue samples, (3) the patients had not undergone prior chemotherapy or radiotherapy, and (4) the total number of samples in the dataset was equal to or greater than 50. Following the application of these stringent criteria, we obtained four datasets [GSE62452 (34), GSE15471 (35), GSE62165 (36), GSE71729 (37)] that met our requirements. To merge and de-batch the four datasets, we utilized the limma package (version 3.50.3) in R. Furthermore, we performed background calibration, normalization, and log2 logarithm conversion of the original data from the four datasets using the affy package (version 1.78.0). In cases where multiple probes recognized the same gene, we calculated the average value to estimate the gene expression. To address any potential batch effects after integrating the datasets, we employed the sva (Version 3.48.0) R package for batch effect removal (38–40).

The single-cell sequencing data were obtained from the GEO database under the registration number GSE155698. The dataset consisted of sequencing results from both tumor tissues and normal tissues, which were extracted and utilized for our analysis. The original data had been preprocessed by the submitter, and the following steps were undertaken: The samples were processed on either the Illumina HiSeq 4000 or NovaSeq 6000 platforms, employing paired-end 50-cycle reads to achieve a sequencing depth of 100,000 reads. The raw data analysis and alignment were performed by the DNA Sequencing Core at the University of Michigan. For data processing, we utilized Cellranger count version 3.0.0 with default settings and an initial estimated cell count of 10,000. Each sample was aligned using the hg19 reference included in the cellranger software.

The transcriptome data and survival data from the TCGA database were acquired through the UCSC Xena online website. The downloaded data had undergone preprocessing, and we removed samples with gene expression values of ‘0’. Paired samples were retained for paired sample analysis. To facilitate further analysis, the Toil technique was employed to transform and standardize the RNA sequencing data into transcripts per thousand bases per million fragments (TPM) format, which was then converted to log2 per million reads. The patients’ characteristics included in the analysis comprised survival time and survival status. Patients under the age of 18 and those with a survival time of less than 30 days were excluded from the analysis.

### 2.2 Obtaining differential genes and module genes based on the GEO expression matrix

To assess the immune infiltration patterns and the differences in immune cell infiltration between tumor tissue and normal tissue, we employed the CIBERSORT method. This approach utilizes linear support vector regression and is based on a known reference dataset (default: LM22) containing gene expression features of 22 immune cell subtypes. We applied the CIBERSORT method to deconvolute the expression matrix of human immune cell subtypes within the combined GEO expression matrix. The resulting immune cell infiltration percentages were visualized using a bar plot, providing an overview of the immune cell composition. Furthermore, we employed box plots to assess whether there were significant differences in the infiltration of each immune cell subtype between the tumor and normal tissue groups. These analyses aimed to identify module genes associated with immune infiltration.

To identify differentially expressed genes (DEGs), we utilized the limma package (version 3.50.3) (41–43). The analysis was performed by comparing the expression levels between different groups, and genes with a p-value less than 0.05 were initially selected as DEGs.

To examine the relationship between gene sets and sample phenotypes, construct regulatory networks connecting gene sets, and identify crucial regulatory genes, we employed the WGCNA method (44, 45). The WGCNA analysis relied on packages such as WGCNA (version 1.71). In the WGCNA process, we utilized a signed network approach. Initially, the Pearson correlation coefficient between pairs of genes was calculated to construct the gene co-expression network. A threshold was applied to identify modules consisting of closely related genes. In constructing the co-expression network, we determined the optimal soft threshold as 6 and the average connectivity as 4.26. The minimum module size was set to 60, and a deepsplit value of 2 was used. The thresholds for module membership (MM) and gene significance (GS) were set at |MM| > 0.9 and |GS| > 0.2, respectively. Subsequently, we correlated the module information with the results obtained from the CIBERSORT immune infiltration analysis (46). This analysis allowed us to identify 22 modules that were associated with immune cell infiltration. Through further screening based on correlation coefficients (R ≥ 0.5) and a significance threshold (P < 0.05), we identified the most relevant module genes associated with Treg cell infiltration.

### 2.3 Preliminary processing of single-cell sequencing data

The comprehensive analysis of single-cell sequencing data was performed using the Seurat package (Version 4.3.0). To ensure the exclusion of low-quality data resulting from cell damage or library preparation failures, we conducted quality control on the single-cell sequencing data according to the following criteria: (1) Cells with less than 500 or more than 6000 expressed genes were excluded; (2) Cells with a unique molecular identifier (UMI) count value less than 1000 were removed, and the top 3% of cells with the highest UMI count were eliminated. (3) Cells with mitochondrial gene expression exceeding 35% of the total gene expression were excluded, and the top 2% of cells with the highest mitochondrial gene expression were removed. (4) The proportion of ribosomal RNA (rRNA) expression in the total gene expression was calculated, and the smallest top 1% and largest top 1% of cells based on rRNA expression were removed.

As this study involved multiple samples, it was necessary to account for experimental variations introduced by different factors. We addressed this by integrating and de-batching the samples using the Harmony package (version 0.1.0). The NormalizeData function was applied to normalize the data, accounting for different cell sequencing depths. The FindVariableFeatures function was used to select 2000 highly variable genes for downstream analysis (33, 47). The ScaleData function transformed gene expression values into z-scores to follow a Gaussian distribution, and the RunPCA function performed initial linear dimension reduction on the single-cell data. To further reduce the data dimensions while preserving important features, we employed the uniform manifold approximation and projection (UMAP) method for final nonlinear dimension reduction (48, 49). This mapping process aimed to capture the maximum data variance in a lower-dimensional space suitable for observation. The FindNeighbors function constructed a K Nearest Neighbor (KNN) network based on Euclidean distance in the principal component analysis (PCA) space (50–52). The edge weights between cells were then adjusted based on the shared overlap (Jaccard similarity) in their local neighborhood to finalize the cell clustering. Cell clusters were determined using the FindClusters function, optimizing the standard modular functionality with a resolution of 0.5. Finally, the Dimplot function was utilized to visualize the effectiveness of cell clustering.

### 2.4 Annotation and reclassification of cell clusters

To ensure the accuracy of cell cluster annotation, we employed various methods for integration. Initially, we performed a preliminary annotation of each cell cluster using the singleR package (version 1.8.1) (53). Subsequently, we utilized the FindAllMarkers method with a significance threshold of P < 0.05 to identify genes that exhibited differential expression between each subgroup and all other subgroups. These differentially expressed genes served as cell markers. To refine the annotation, we manually reviewed relevant literature and consulted online databases such as CellMarker and BMC Genome Biology (54). This comprehensive approach allowed us to annotate each cell subgroup accurately. In order to distinguish between benign and malignant cells in the tumor microenvironment, we employed the copykat program (version 1.0.8) to determine the genome copy number distribution of individual cells. By integrating Bayesian techniques and hierarchical clustering, copykat enabled us to classify cells into diploid normal cells or aneuploid tumor cells. The copykat program utilized a Gaussian Mixture Model (GMM) definition model, assuming that a cell’s gene expression was a mixture of three Gaussian models: amplification, deletion, and neutral state. Cells that had the neutral gene accounting for at least 99% of the expressed genes were classified as high-confidence diploid cells. Consequently, we categorized the cells into diploid cells (benign) and aneuploid cells (tumor). Since Treg cells were part of the T cell clusters, we further conducted an in-depth analysis of Treg cells. We extracted the T cell clusters, performed data normalization, identified highly variable genes, applied dimensionality reduction techniques such as centralization, PCA, and UMAP, and identified cell clusters. Finally, we annotated the Treg cells within the identified clusters for further analysis.

### 2.5 Quasi-timing analysis and cell communication analysis

To understand the differentiation trajectory of T cells and the evolution of cell subtypes during development, we utilized the monocle package (version 2.22.0) to perform pseudo-time series analysis on T cell subsets based on their gene expression changes over time. After estimating size factors and dispersions, we applied the detect_genes function to filter out low-quality cells, setting the expression threshold to 0.1. Next, we selected the top 200 clusters of differentially expressed genes and used the DDRTree method within the reduced dimension function to reduce the data dimensionality. This enabled us to calculate the development time, infer the trajectory, and sort the cells based on their pseudo-time ordering. The results were visualized in the form of a tree diagram, representing the inferred differentiation trajectory. To identify key genes involved in the inferred development trajectory, we employed the beam statistical method. This involved analyzing the cell data after pseudo-time sorting and specified nodes, calculating the contribution value of each gene during cell development. The key genes were then ranked and outputted based on their contribution value. These genes were considered differential genes that played a crucial role in cell development and differentiation.

To investigate the cell communication between Treg cells and other cells in the tumor microenvironment, as well as the specific activated cell signaling pathways, we conducted an analysis using the cellchat package (version 1.1.3). The analysis was based on the CellChatDB database, which encompasses 1939 validated molecular interactions in the human body. These interactions comprise 61.8% paracrine/autocrine signaling interactions, 21.7% extracellular matrix (ECM)-receptor interactions, and 16.5% cell-cell contact interactions. In our analysis, we identified overexpressed ligands or receptors within a specific cell population and subsequently identified overexpressed ligand-receptor interactions when both ligands and receptors were found to be overexpressed. This allowed us to infer cell state-specific communication. CellChat assigned a probability value to each contact and performed a permutation test to determine biologically significant cell-cell communication. The likelihood of cell communication was simulated by combining gene expression data with known information about the interaction between signal ligands, receptors, and their cofactors, using the law of mass action. Based on the inferred cell-cell communication network, we employed various visualization techniques and quantitative analysis to visualize the major senders (sources) and receivers (targets) in the signaling pathways. This approach enabled us to identify the signals that significantly influenced the outgoing or incoming signals of specific cell populations, providing insights into the key players involved in cell communication within the tumor microenvironment.

### 2.6 Acquisition and verification of Treg cell-related genes

From the above analyses, we obtained four gene sets: the differential genes identified from the GEO expression matrix, the module genes identified through WGCNA, the Treg cell marker genes identified from single-cell subgroup analysis, and the differential genes implicated in T cell differentiation and development from pseudo-temporal analysis. To identify Treg cell-related genes, we performed an intersection of these four gene sets. The results of the intersection were visualized using a Venn diagram. The obtained Treg cell-related genes were further validated and screened in the TCGA database. We examined the association between these TRGs and the survival of pancreatic cancer patients using survival analysis methods, enabling us to identify genes that were significantly associated with patient prognosis. Furthermore, we evaluated the diagnostic efficacy of Treg cells for pancreatic cancer patients in the TCGA cohort using receiver operating characteristic curve (ROC) analysis (55). Genes that exhibited an area under the ROC curve (AUC) greater than 0.7, along with their prognostic relevance, were selected as the most critical genes within the Treg cell gene set. To verify the expression of these TRGs in pancreatic cancer tissues, we accessed the human protein atlas database, which provided immunohistochemistry data for further validation. Overall, this comprehensive approach allowed us to identify a set of Treg cell-related genes associated with prognosis and diagnostic potential in pancreatic cancer.

### 2.7 Drug sensitivity analysis guided by TRGs

The drug sensitivity analysis was conducted using the oncoPredict package (version 0.2) developed by Danielle Maeser et al. in 2021 (56). This algorithm leverages three functions: GLDS, calcPhenotype, and IDWAS, as well as two databases, namely the Cancer Therapeutics Response Portal (CTRP) and the Genomics of Drug Sensitivity in Cancer (GDSC). The GLDS function was employed to identify markers in cell lines, while the calcPhenotype function utilized large-scale gene expression and drug screening data to establish a ridge regression model. This model was then applied to new gene expression data to predict clinical chemotherapy response. The IDWAS function was utilized to measure drug-gene interactions and identify biomarkers associated with drug response. This function incorporated drug response data along with somatic mutation or copy number variation (CNV) data obtained from population sequencing. The expression score of TRGs was calculated as the average sum of their expression levels. In the context of pancreatic cancer, patients whose TRGs expression score exceeded the median score of all patients were classified as high-risk, while those below the median were considered low-risk. This approach facilitated the identification of patients who were more likely to exhibit drug resistance or sensitivity, providing valuable information for personalized treatment strategies.

### 2.8 Identification of key signaling pathways regulating two kinds of Treg cell different survival outcomes by hdWGCNA

The implementation of WGCNA for single-cell data utilized the hdWGCNA package (version 0.2.2) developed by Sam Morabito et al. (57, 58). This package specifically catered to the analysis of single-cell sequencing data, allowing for the construction of co-expression networks across multi-scale cells and spatial hierarchies. To begin, we established a Seurat object to facilitate the WGCNA process. The hdWGCNA package employed the KNN algorithm to identify similar cell groups that could be aggregated. The average or sum expression of these cells was then calculated, resulting in a low sparse metacell gene expression matrix. The SetDatExpr function was utilized to specify Treg cells for constructing the expression matrix. Next, we performed parameter scans using the TestSoftPowers function to determine the optimal soft power threshold for constructing the co-expression network. By evaluating the resulting network topology at different power values, we selected the soft power threshold that retained a strong gene-gene correlation adjacency matrix while removing weak connections. In this study, a scale-free topology model was chosen, with a minimum soft power threshold set at 0.8 or higher. The ConstructNetwork function was employed to establish the co-expression network based on the optimal soft threshold. Subsequently, the ModuleEigengenes function was utilized to calculate the module feature genes (ME) by performing principal component analysis (PCA) on a subset of the gene expression matrix specific to each module (59).

Additionally, the ModuleExprScore function, using either the Seurat or UCell algorithm, was used to compute the central gene feature score for each module. To visualize the correlation between modules, the ModuleCorrelogram function was applied, considering the hME, ME, or hub gene scores. The GetModuleTraitCorrelation function was utilized to screen for important modules based on their correlation coefficients and P-values in relation to specific traits or characteristics of interest. Through these steps, the hdWGCNA package facilitated the identification of robust modules of interconnected genes in single-cell sequencing data, allowing for comprehensive WGCNA analysis and exploration of gene co-expression patterns.

### 2.9 Polymerase chain reaction

For the PCR analysis, tumor tissues from 24 patients with pancreatic cancer were obtained from the First Affiliated Hospital of Soochow University. The study protocol was approved by the Ethics Committee of the First Affiliated Hospital of Soochow University and adhered to the principles of the Helsinki Declaration. RNA extraction was performed using RNAeasy reagent (Vazyme, Nanjing, China), and reverse transcription was carried out using the HiScript III first-strand cDNA synthesis kit (Nanjing, China) as per the manufacturer’s instructions. Real-time PCR was performed on the ABI StepOne PlusTM real-time PCR system using SYBR^®^ Green for Master Mix (Vazyme, Nanjing, China) (60). The relative mRNA expression was calculated in triplicate using the 2-ΔΔCt method (61, 62).

As the relative mRNA expression levels of PDCD1 and CTLA4 measured by PCR were skewed, the correlation analysis between TRGs and immune checkpoints was performed using Spearman’s rank correlation analysis. See **Table S1** for primer sequence.

### 2.10 Hematoxylin-eosin staining

Use xylene solution and gradient alcohol for dewaxing of the sections. After immersing in hematoxylin staining solution for 10 minutes, remove and rinse with deionized water. Perform differentiation using 1% hydrochloric acid alcohol solution, immerse for 5 seconds, then remove and rinse with deionized water. After counterstaining with 1% ammonia water for 1 minute, rinse with deionized water. Finally, perform eosin staining. Immerse in eosin staining solution for 1 minute, then rinse with deionized water. Sequentially immerse in gradient ethanol (70%, 80%, 90%) for 20 seconds each, followed by absolute ethanol for 1 minute, and xylene solution for 5 minutes (repeat this step twice). After removing the sections, dry them and observe after mounting.

### 2.11 Immunohistochemical staining

The prepared wax blocks were dewaxed at 60°C, followed by sequential immersion in pure xylene and gradient ethanol (100%, 95%, 80%, 75%), and soaked in 3% hydrogen peroxide for 10-15 minutes. Subsequently, perform antigen retrieval, block the antigen, and incubate with primary and secondary antibodies separately. Employ DAB staining, monitor the progress under an optical microscope, and then counterstain using hematoxylin. After each step, rinse with PBS (63).

### 2.12 Immunofluorescence staining

Immunofluorescence analysis involved collecting paraffin sections of paired pancreatic cancer tissues and adjacent normal tissues from the First Affiliated Hospital of Soochow University. The sections underwent dewaxing, hydration, antigen retrieval, and blocking procedures (64). FOXP3 (Servicebio, Wuhan, China) was detected using immunofluorescence staining. The number of CD4/FOXP3-positive cells in cancer and adjacent tissues was counted using fluorescence microscopy (65).

### 2.13 Statistical methods and online database

All data processing in this study was conducted using R (version 4.1.3). The differential analysis for all experimental groups and comparison groups was performed using the Wilcoxon rank sum test (66, 67). Correlation analysis of public databases was conducted using the Pearson correlation method. For the correlation analysis of PCR data, the Spearman correlation method was applied (68). A significance level of 0.05 was used for all analyses, and the corresponding difference multiples and correlation coefficients were specified for each step. The P-values were adjusted using the default Benjamini-Hochberg correction method.

GEO: https://www.ncbi.nlm.nih.gov/geo/
TCGA: https://www.cancer.gov/about-nci/organization/ccg/research/structural-genomics/tcga/
UCSC Xena: https://xenabrowser.net/datapages/
CIBERSORTx: https://cibersortx.stanford.edu/
CellMarker: http://xteam.xbio.top/CellMarker/
BMC Genome Biology: https://genomebiology.biomedcentral.com/
The human protein atlas: https://www.proteinatlas.org/
Cancer Therapeutics Response Portal: http://portals.broadinstitute.org/ctrp/
Genomics of Drug Sensitivity in Cancer: https://www.cancerrxgene.org/
Ensemble: http://asia.ensembl.org/index.html

## 3 Results

### 3.1 CIBERSORT deconvolution demonstrated the infiltration of immune cells in the tumor microenvironment of patients with pancreatic cancer

**Figure 1:**
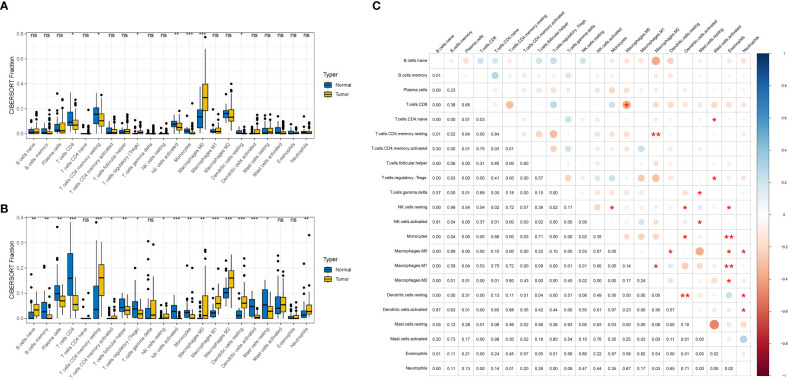
Analysis of immune cell infiltration in patients with GEO large sample data. **(A, B)** Boxplot qualitatively analyzed the difference of infiltration of 22 immune cells in tumor tissues and normal tissues. **(A)** GSE15471, **(B)** GSE71729, **(C)** Heatmap of co-expression relationship among 22 immune cells. Red represents positive correlation and blue represents negative correlation. ns: *P* < 0.1. *: *P* < 0.05. **: *P* < 0.01. ***: *P* < 0.001.

**Supplementary Figure 1** showed the flow of this study. After merging four samples from GEO, we obtained an expression matrix comprising 530 samples, including 159 normal tissues and 371 pancreatic cancer tissues. Immune infiltration analysis of the expression matrix revealed the presence of 22 immune cell types infiltrating the pancreatic cancer microenvironment. To provide a clearer visualization of immune cell infiltration, a qualitative analysis was conducted. The boxplot demonstrated varying degrees of infiltration for several immune cell types between pancreatic cancer tissues and normal tissues, such as B cells naive, B cells memory, T cells CD8, T cells CD4 naive, NK cells resting, NK cells activated, Monocytes, among others. However, no significant difference in Treg cell infiltration between the tissues was observed. However, upon conducting immune infiltration analysis on the four datasets separately, we observed differences in the infiltration of Treg cells between the two groups in GSE15471 and GSE71729. This suggests a potential association between the infiltration of Treg cells and pancreatic cancer (**Figures 1A, B**). The immune infiltration correlation heat map revealed a positive correlation between Treg cell infiltration and Macrophages M0, while no significant correlations were observed with the infiltration of other cell types (**Figure 1C**).

### 3.2 Differential analysis and functional enrichment revealed unique genetic and functional changes in pancreatic cancer

**Figure 2:**
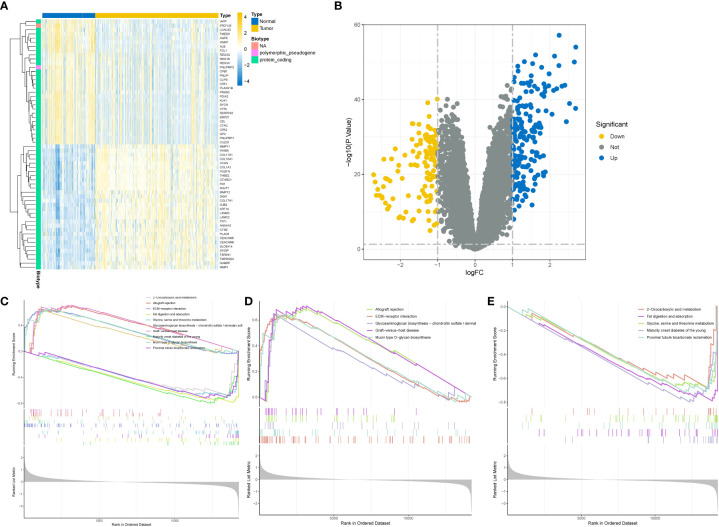
Difference and enrichment analysis of normal and tumor tissues in GEO cohort. **(A)** Differential gene heatmap, the left side is the normal group, the right side is the tumor group. **(B)** Differential gene volcano map, the horizontal axis is the difference multiple takes log2 logarithm, the vertical axis is the *P* value takes - log10 logarithm. Blue represents high expression, yellow represents low expression. **(C–E)** Enrichment analysis. **(C)** All differential gene enrichment analysis results. **(D)** The results of up-regulated differential gene enrichment analysis. **(E)** Results of down-regulated differential gene enrichment analysis.

To minimize the potential influence of different batches, sequencing personnel, and machines on the analysis, we initially standardized the expression matrix. In order to retain a substantial number of genes, we applied a threshold of P < 0.05 for the initial screening. Consequently, a total of 10,759 genes passed this screening, including 4,696 up-regulated genes and 6,163 down-regulated genes. The heatmap visually depicted the top 30 up-regulated and down-regulated genes with the highest logFC values (**Figure 2A**), while the volcano plot displayed the genes with logFC > 1 (**Figure 2B**). Through gene set enrichment analysis (GSEA), we observed significant up-regulation of processes such as allograft rejection, ECM-receptor interaction, and Mucin type O-glycan biosynthesis in pancreatic cancer tissues compared to normal tissues. Conversely, down-regulated functions included 2-Oxocarboxylic acid metabolism, Fat digestion and absorption, as well as Glycine, serine, and threonine metabolism (**Figures 2C–E**). The results of the enrichment analysis not only further validated the accuracy of the differential analysis but also provided insights into the significantly altered biological functions and signaling pathways in pancreatic cancer tissues.

### 3.3 WGCNA identified yellow module as most relevant to Treg cell infiltration

**Figure 3:**
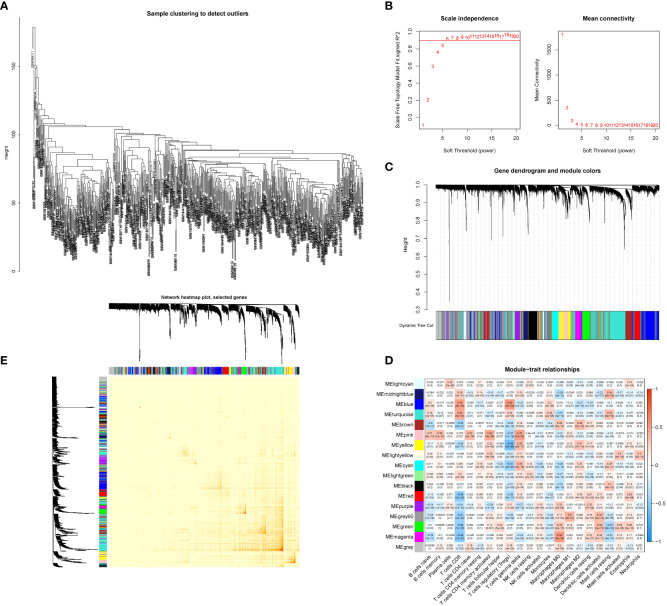
Identification of Treg cell related mRNA by weighted gene co-expression network (WGCNA). **(A)** Sample clustering tree diagram, cluster analysis of all samples, the tree diagram shows that there is no significant outlier in the sample. **(B)** Determine the optimal soft thresholding or power to make the constructed network more consistent with the scale-free topology. Left figure: scale-free fit index (y-axis) under different soft thresholds (x-axis). The red line represents the subjectively selected scale-free fitting index value, which is 0.9 in this study. **(C)** Construct a co-expression network based on the optimal soft threshold, and divide the genes into different modules to draw a gene clustering tree. The upper part is the hierarchical clustering tree of genes, and the lower part is gene module, namely network module. **(D)** Calculate the correlation and significance between the module and 22 kinds of immune cell infiltration, and draw a correlation heat map. The first-row number in each module is the correlation coefficient, and the second-row number is the *P* value. Red represents positive correlation; blue represents negative correlation. **(E)** Drawing the correlation heat map between genes based on topological overlap matrix. The darker the color, the stronger the interaction between genes. The diagonal represents the interaction between genes within the module, and the color is the deepest.

We performed correlation coefficient calculations and found that a correlation coefficient greater than 0.9 (with a soft cutoff of 6) indicated a strong and suitable basis for constructing multiple gene modules (**Figures 3A, B**). Utilizing the correlation and adjacency matrices of gene expression profiles, we constructed a topological overlap matrix (TOM). The resulting gene cluster tree was displayed in **Figure 3C**. Subsequently, we applied a hierarchical average linkage clustering approach and TOM to identify gene modules within each gene network. The heatmap visualized the identified gene modules, and by employing a dynamic tree-cutting technique, we identified a total of 17 gene modules (**Figures 3D, E**). Based on the criteria of a correlation coefficient (R) ≥ 0.5 and P < 0.05, we found that the yellow module exhibited a strong negative correlation with Treg cell infiltration (r = -0.58, P = 2e-49), while displaying weak correlations with other immune cell infiltrations. Finally, we extracted 1,283 genes from the yellow module for further analysis.

### 3.4 Single-cell sequencing data revealed unique tumor microenvironment features of pancreatic cancer

**Figure 4:**
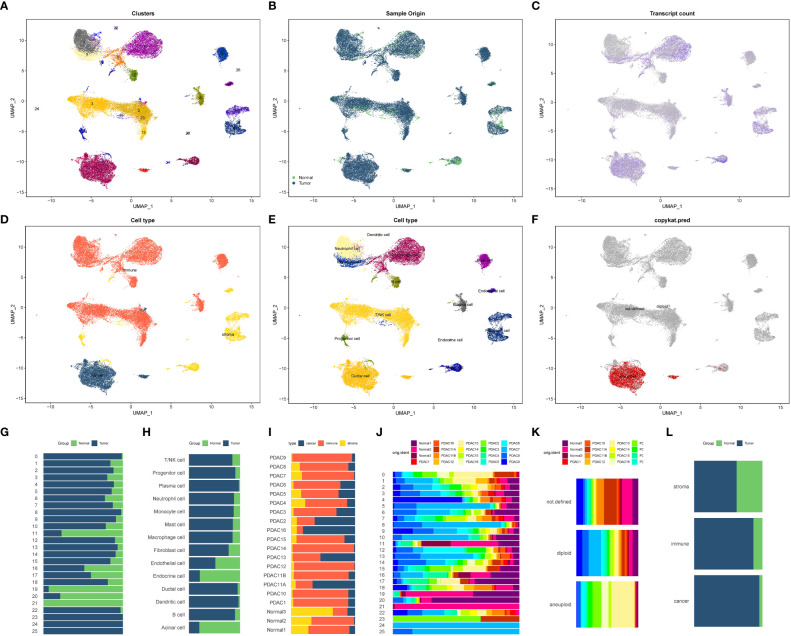
Cell annotation and proportion display of 20 single-cell sequencing samples **(A–F)** 20 samples of all cell UMAP dimension reduction map. **(A)** Cluster distribution of 26 cells. **(B)** Cell distribution in normal and tumor tissues. **(C)** Each cell gene expression showed. **(D)** All cells were classified based on tumor cells, stromal cells and immune cells. **(E)** Distribution of all cell types after cell annotation. **(F)** Aneuploid cells (tumor cells) based on copykat recognition. **(G–L)** The proportion of each cell component in different tissues. **(G)** Proportion of 26 cell clusters in normal and tumor tissues. **(H)** The proportion of 14 cells in normal tissues and tumor tissues. **(I)** The proportion of three cell components (immune cells, tumor cells and stromal cells) in 20 tissue samples. **(J)** Proportion of 26 cell subsets in 20 samples. The proportion of tumor cells, non-tumor cells and unrecognized cells identified by **(K)** Copykat method in 20 tissues. **(L)** The proportion of three cell types (immune cells, tumor cells and stromal cells) in normal tissues and tumor tissues.

We obtained 20 single-cell sequencing samples of pancreatic cancer and normal tissues from the GEO database, encompassing 55,339 cells and 24,904 genes. After filtering out low-quality cells, we obtained a final dataset of 40,084 cells and 24,904 genes, including 3 normal tissues and 17 pancreatic cancer tissues. Utilizing the FindVariableFeatures function, we selected 2,000 highly variable genes for subsequent analysis. Principal component analysis (PCA) was performed for dimension reduction, and the first 30 dimensions were selected for UMAP dimension reduction. Consequently, we identified 26 distinct cell subsets (**Figure 4A**). The expression patterns of these cell subsets in different tissue types were illustrated in **Figure 4B**, and **4C** depicted the distribution of gene expression across different cell types. **Figure 4D** showcased the distribution of stromal cells, tumor cells, and immune cells in pancreatic cancer tissues based on specific marker genes.

By employing various annotation methods, we successfully annotated the identified cell types (**Figure 4E**). In total, we identified 14 cell types in this study. The T/NK cell marker genes included CD3D, CD4, CD8A, and CD7, while B cell marker genes comprised MS4A1 and CD19. Plasma cell marker genes consisted of CD38 and CD27, and mononuclear/macrophage marker genes included CD68, CD163, ITGAM, and CD14. Neutrophil marker genes were S100A8, FCGR3B, MNDA, and CXCR2. Mast cell marker genes were SLC18A2, ENPP3, FCER1A, and ACSL4. Dendritic cell marker genes were PTCRA and GZMB. Progenitor cell marker genes encompassed CD34, KDR, ASPM, and CDKN3. Acinar cell marker genes were PRSS1, ALB, AQP8, and AMY2A. The duct cell marker gene was CD133, and endocrine cell marker genes included SLC30A8, GCG, CRYBA2, and TTR. Fibroblast cell marker genes comprised FGF7, ACTA2, and COL11A1. Vascular endothelial cell marker gene was VWF, stromal cell marker gene was MME, immune cell marker gene was PTPRC, and epithelial cell marker gene was EPCAM (**Supplementary Figure 2**). Copykat analysis revealed that the primary source of malignant cells in the pancreatic cancer tumor microenvironment was ductal cells, followed by acinar cells (**Figure 4F**). The proportions of different subsets and cell types in various tissues and samples were presented in **Figures 4G–L**.

### 3.5 T/NK cell subsets reclassification and pseudo-sequential analysis revealed Treg differentiation related genes

**Figure 5:**
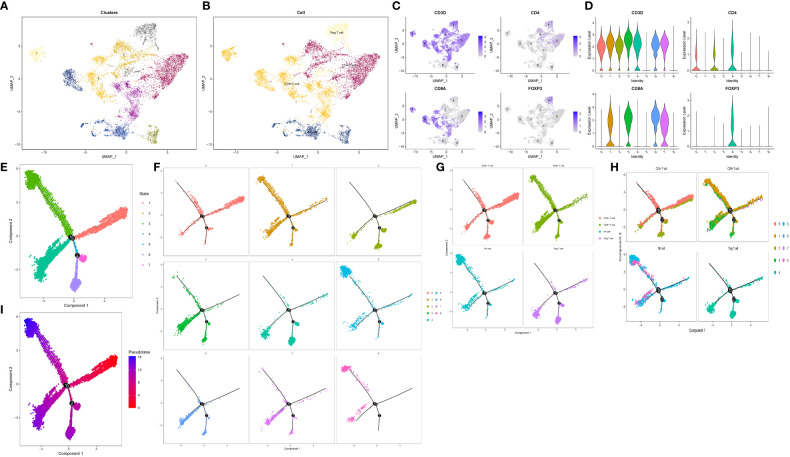
Reclassification and pseudo-timing analysis of T/NK cell cluster**. (A, B)** Reclassification of T/NK cell cluster. **(A)** The display of 9 cell subsets after UMAP dimensionality reduction. **(B)** Four cell types after annotation. **(C, D)** Characteristic distribution of marker genes. **(E–I)** Cell trajectory analysis revealed the developmental trajectories of various subgroups and cell types. **(E)** Trajectory analysis All branches are displayed. **(F)** Trajectory analysis according to 9 cell subsets. **(G)** According to the trajectory analysis of 4 cell types. **(H)** Trajectory analysis according to 4 cell types and 9 cell subsets. **(I)** Differentiation trajectory of cell differentiation maturity.

To investigate Treg cells and explore their transitional relationship with T/NK cells, we isolated and reclassified the T/NK cell cluster. Using UMAP dimension reduction with 15 dimensions, we identified nine distinct cell clusters (**Figure 5A**). Subsequently, we subdivided the T/NK cell clusters into CD4+ T cells, CD8+ T cells, Treg cells, and NK cells based on specific cell marker genes such as CD4, CD8A, FOXP3, and NKG7 (**Figures 5B–D**). Quasi-temporal analysis revealed the presence of three pivotal branching points and seven branches during the redevelopment and differentiation of the T/NK cell cluster (**Figure 5E**). The distribution of cell types along the cell trajectories within different subgroups was displayed in **Figures 5F–H**. By considering biological significance and statistical algorithms, we determined the starting point of the quasi-temporal trajectory (69). As cells moved further away from the starting point, their developmental maturity increased (**Figure 5I**). Treg cells infiltrating the pancreatic cancer microenvironment exhibited low expression of CD4 molecules and displayed a mature differentiation state, primarily derived from CD4+ T cells. Furthermore, Treg cells exhibited higher expression of genes such as TNFRSF4, CTLA4, RTKN2, compared to other T cells and NK cells. Through Beam analysis, we identified 1,235 genes that displayed gradual increases or decreases in expression during cell differentiation, suggesting their crucial role in determining cell state transitions.

### 3.6 Cell communication analysis identified the pathways and optimal ligand-receptor of Treg cell

**Figure 6:**
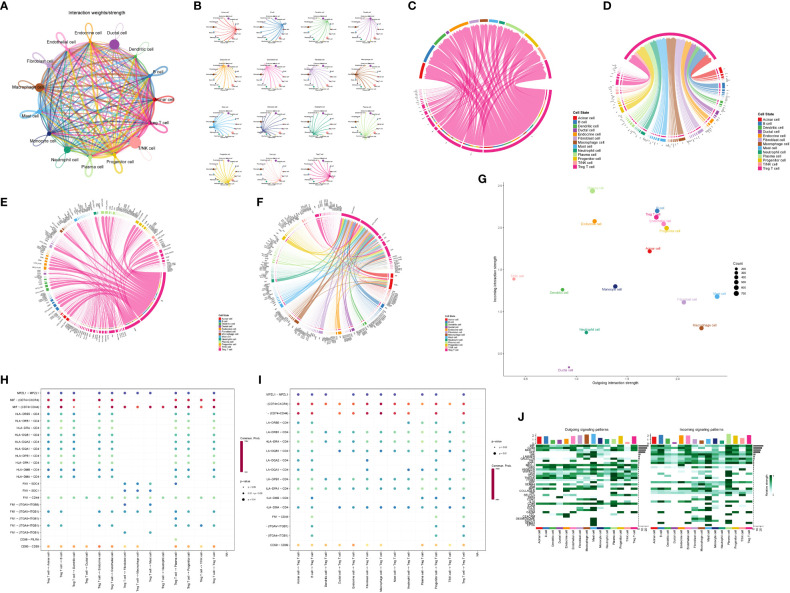
Analysis of communication between Treg cell and other cells in pancreatic cancer microenvironment. **(A)** Integrated map of the overall communication intensity of each cell in pancreatic cancer tissues. **(B)** Show the overall communication intensity of each cell in pancreatic cancer tissues. **(C, D)** Treg cells are important signaling pathways involved as signal senders and receivers. **(E, F)** Treg cells are important ligands-receptors in important signaling pathways involved in signal senders and receivers. **(G)** Overall strength of Treg cells as signal receivers (vertical axis) and transmitters (horizontal axis). **(H, I)** Treg cells are important ligand-receptors in the five most important signaling pathways involved by signal senders and receivers. **(J)** Intensity demonstration of all cells as signal receivers and transmitters in different pathways.

The analysis of cell communication revealed the extent and pathways through which Treg cells interacted with other cells within the pancreatic cancer microenvironment. We identified a total of 38 significantly altered signaling pathways in the microenvironment. Treg cells displayed close associations with various cell types in the microenvironment (**Figures 6A, B**). Notably, Treg cells were prominently involved in five pathways: the CD99 pathway, FN1 pathway, MHC-II pathway, MIF pathway, and MPZ pathway (**Figures 6C, D**). Within the CD99 pathway, the CD99-CD99 receptor interaction exhibited the greatest contribution. In the FN1 pathway, the FN1-CD44 receptor interaction played a major role. The HLA-DRA-CD4 ligand-receptor interaction made the most significant contribution to the MHC-II pathway. Within the MIF pathway, the MIF-CD74 + CD44 receptor interaction was highly influential. Finally, the MPZ1-MPZ1 pathway demonstrated the highest contribution to the MPZ pathway (**Figures 6E, F**, **Supplementary Figures 3**–**7**). **Figures 6G–J** visualized the roles of Treg cells and other cell types as both signal transmitters and receivers within these five pathways.

### 3.7 CASP4, FYN, TOB1, CLEC2B were identified as TRGs

**Figure 7:**
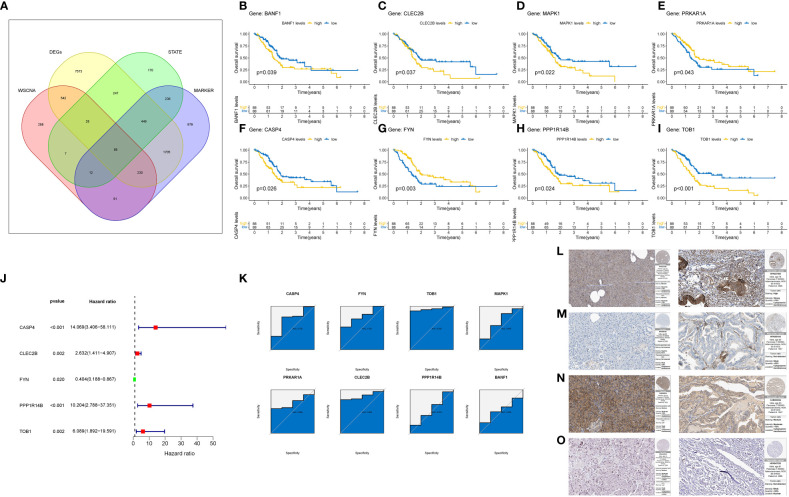
Identification and Validation of TRGs. **(A)** Venn diagram shows the number of intersection genes of four gene sets. **(B–I)** Verify the 8-gene K-M survival curve closely related to the prognosis of pancreatic cancer patients in the TCGA cohort. **(J)** COX regression model of 8 Gene. **(K)** ROC analysis of 8 Gene. **(L–O)** Final identification of 4 gene immunohistochemical results. The left is normal tissue and the right is tumor tissue. **(L)** CASP4. **(M)** CLEC2B. **(N)** FYN. **(O)** TOB1.

Following the aforementioned analyses, we obtained 85 genes based on four gene sets: differentially expressed genes between pancreatic cancer and normal tissues, Treg cell-related genes identified by WGCNA, marker genes of Treg cells in single-cell sequencing, and differentiation-related genes from pseudo-temporal analysis (**Figure 7A**). To further refine the gene selection and identify the most crucial genes, we validated and screened these 85 genes using the TCGA cohort. Kaplan-Meier (K-M) survival analysis narrowed down the selection to 12 genes that exhibited a significant correlation with the survival of pancreatic cancer patients (**Figures 7B–I**). Subsequently, we applied the COX regression model and conducted ROC analysis to identify the final set of tumor-related genes (TRGs), which included CASP4, FYN, TOB1, and CLEC2B (**Figures 7J, K**). Immunohistochemical expression analysis of these four genes in both normal and pancreatic cancer tissues further confirmed our findings (**Figures 7L–O**).

### 3.8 Patients in different risk groups had different immune checkpoint expression and potentially effective drugs

**Figure 8:**
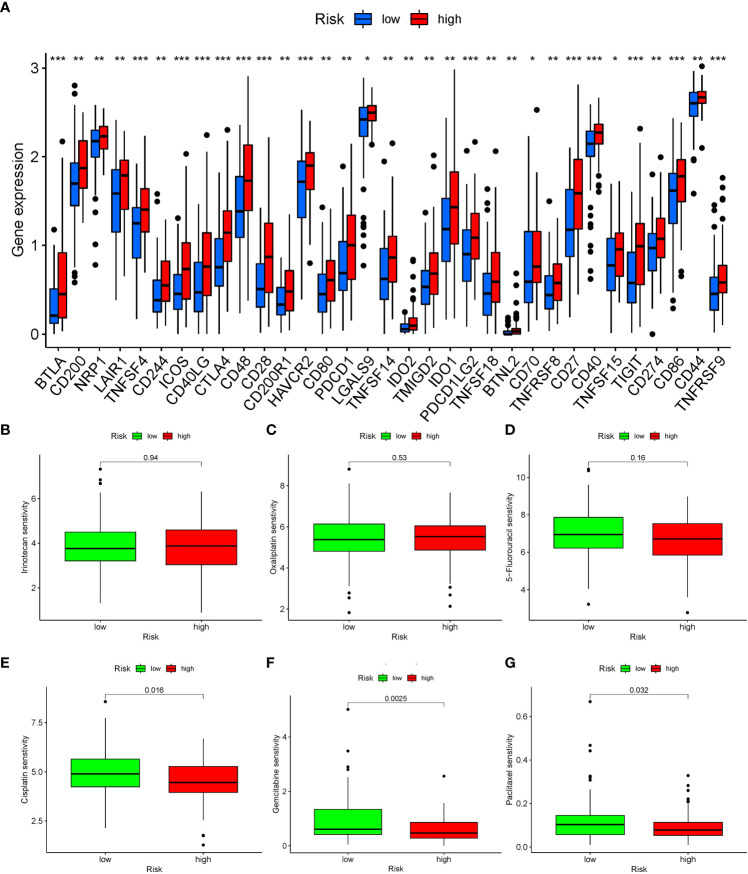
Analysis of immune checkpoint expression and drug sensitivity in patients with different risk groups. **(A)** The expression of immune checkpoints in patients with different risk groups, blue represents the low-risk group, red represents the high-risk group. *: *P* < 0.05, **: *P* < 0.01, ***: *P* < 0.001. **(B–G)** Five drugs were shown to have significant differences in sensitivity between the two groups of patients. **(B)** Irinotecan. **(C)** Oxaliplatin. **(D)** 5-Fluorouracil. **(E)** Cisplatin. **(F)** Gemcitabine. **(G)** Paclitaxel.

Immune checkpoints represented a class of regulatory mechanisms involved in suppressing the function of human immune cells. These checkpoints were frequently overexpressed in the tumor immune microenvironment, leading to immune evasion and inhibition of the body’s anti-tumor immune response. In our analysis of the immune microenvironment in patients with high expression of TRGs, we observed a significant upregulation of immune checkpoints in pancreatic cancer. Several immune checkpoints, including TNFSF4, ICOS, CTLA4, and PDCD1, have been identified as playing crucial roles in the progression of pancreatic cancer (**Figure 8A**).

Through OncoPredict drug sensitivity analysis, we identified potential therapeutic drugs for clinical treatment of pancreatic cancer based on the TRGs. Gemcitabine, cisplatin and paclitaxel demonstrated distinct therapeutic effects based on the expression levels of TRGs in patients. Notably, there were no discernible differences in the sensitivity to conventional chemotherapy drugs such as irinotecan, oxaliplatin, and 5-Fluorouracil for the treatment of pancreatic cancer (**Figures 8B–G**).

### 3.9 Identification of the HIF-1 signaling pathway involved in the transformation of Treg cell clusters by hdWGCNA

**Figure 9:**
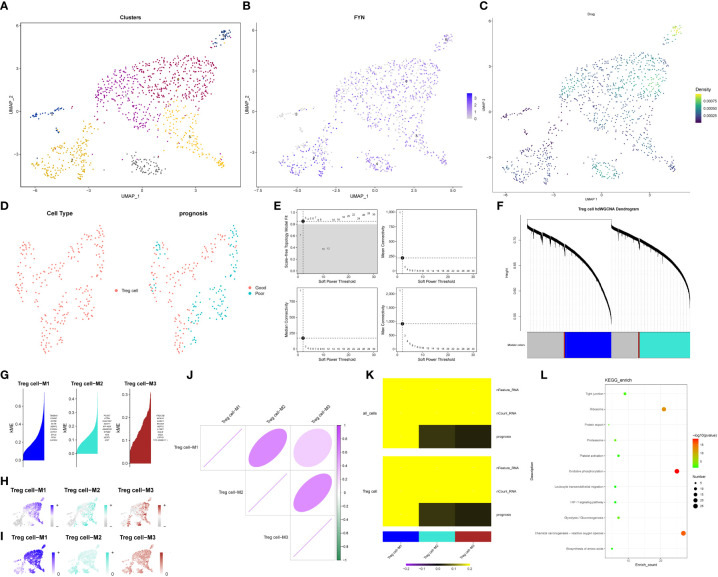
hdWGCNA identifies key signaling pathways regulating the exchange between Treg1 and Treg2. **(A)** Treg cells reclassification dimension reduction diagram. **(B)** The expression level of FYN in Treg cells. **(C)** The results of single cell level enrichment analysis of three genes (CASP4, TOB1, CLEC2B) based on Ucell package. **(D)** Use hdWGCNA to obtain metacell from the Seurat object. **(E)** The scale-free topology model was selected to fit the lowest soft power threshold greater than or equal to 0.8, which made the constructed network more consistent with the scale-free topology. **(F)** The co-expression network was constructed based on the optimal soft threshold, and the gene clustering tree was drawn after genes were divided into different modules. The upper part was the hierarchical clustering tree of genes, and the lower part was gene module, namely network module. **(G)** Feature gene-based connectivity (kME) for each gene was calculated in co-expression network analysis to identify highly connected genes (hub genes) within each module. **(H)** Based on the UCell algorithm, the gene scores of each module gene were calculated. **(I)** Based on UCell algorithm, the gene scores were calculated for central genes of each module. **(J)** The correlation between modules based on Pearson correlation analysis. **(K)** The correlation heatmap drawn by the PlotModuleTraitCorrelation in the hdWGCNA package. **(L)** The result of KEGG enrichment analysis of Module 1 core gene.

Based on the identified TRGs, we investigated the dual role of Treg cells in pancreatic cancer and their impact on patient prognosis. Treg cells were extracted from the T/NK cell cluster using the marker “CD4+ FOXP3.” These Treg cells were then reclassified into seven distinct clusters (**Figure 9A**). Our goal was to identify Treg cell clusters associated with either favorable (Treg1) or poor (Treg2) prognosis. To achieve this, we examined the expression of FYN, which positively correlated with prognosis, and the enrichment scores of three genes (CASP4, TOB1, CLEC2B), which negatively correlated with prognosis. Our analysis revealed widespread expression of FYN in all clusters except cluster 5 (**Figure 9B**). Additionally, clusters 0, 4, and 6 exhibited higher enrichment scores for the three genes of interest (**Figure 9C**). Consequently, we designated clusters 0, 4, and 6 as Treg2 due to their higher three-gene enrichment scores, while the cluster with lower three-gene enrichment scores and high FYN expression was labeled as Treg1. Next, we performed hdWGCNA analysis on the two Treg cell clusters (**Figure 9D**). By selecting an optimal soft threshold of 2, we constructed a co-expression matrix for the single-cell transcriptome (fraction = 0.05) (**Figure 9E**). The TestSoftPowers function (networkType = ‘signed’) was then employed to perform parameter scans across various soft power thresholds (range from 1 to 30). Merging the module similarities yielded three modules (**Figures 9F, G**). The enrichment of all module genes and core genes in Treg cells was presented in **Figures 9H, I**, respectively. **Figure 9J** displayed the correlation between the three modules, revealing that module 1 (Treg cell-M1) exhibited the strongest association with prognosis. We extracted the core genes of module 1 and performed KEGG enrichment analysis, which indicated a high enrichment score in the HIF-1 signaling pathway (P < 0.05) (**Figures 9K, L**). This finding suggested that the HIF-1 signaling pathway plays a critical role in the transition between the two Treg cell clusters, ultimately influencing the anti-tumor immune response in patients with pancreatic cancer and leading to divergent prognosis outcomes.

### 3.10 Further validation of the correlation between four TRGs and pancreatic cancer *in vitro*

**Figure 10:**
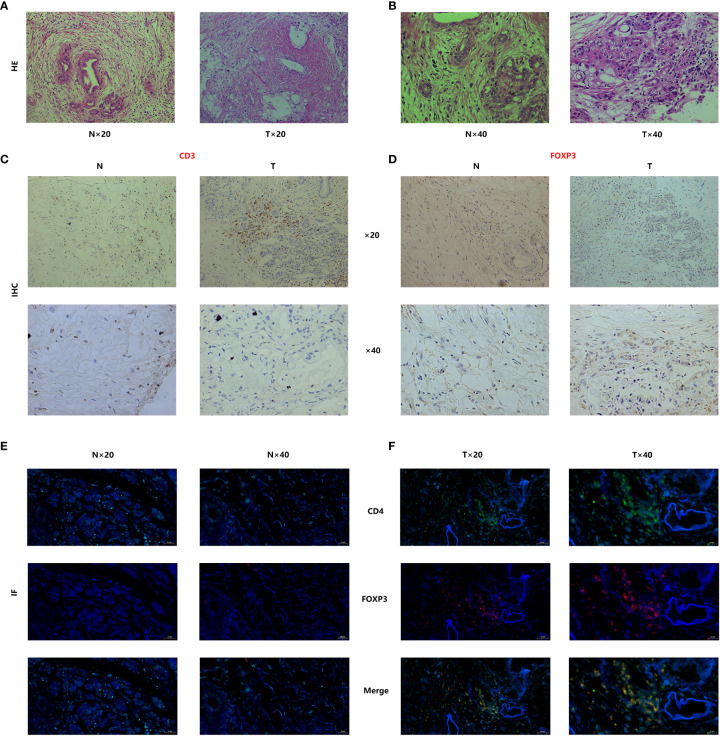
Tissue staining has demonstrated significant infiltration of Treg cells in the tumor microenvironment of pancreatic cancer. **(A, B)** Histopathological staining (Hematoxylin and Eosin staining) results of pancreatic cancer and adjacent normal tissues. **(C, D)** CD3-FOXP3 immunofluorescence staining of pancreatic cancer tissues and adjacent normal tissues. **(E, F)** Immunofluorescence co-staining results of CD4/FOXP3 in pancreatic cancer and adjacent normal tissues.

**Figure 11:**
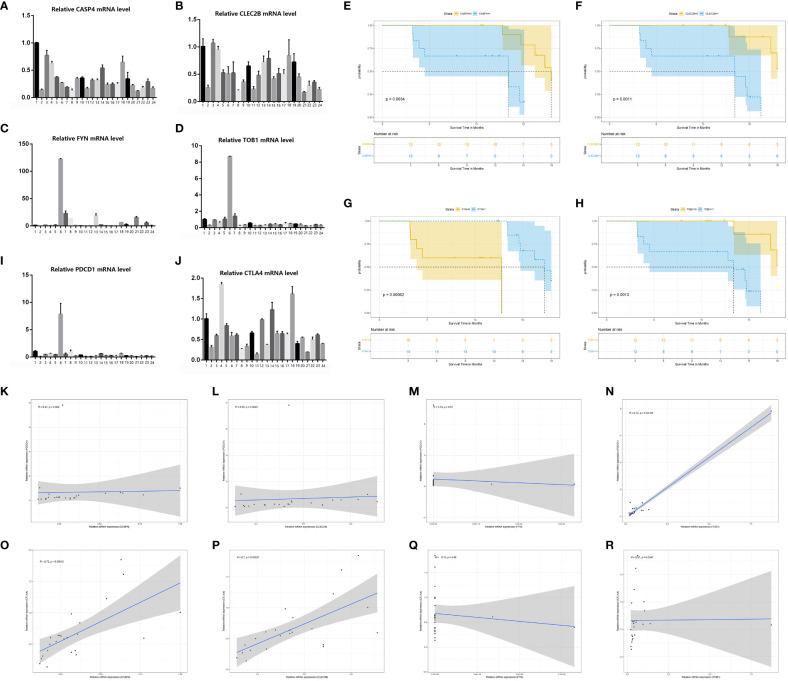
PCR confirmed the results of bioinformatics analysis. **(A–D)** PCR results of four genes (CASP4, FYN, CASP4 and TOB1) in 24 pancreatic cancer tissue samples. **(E–H)** Survival analysis of four genes (CASP4, FYN, CASP4 and TOB1). **(I, J)** PCR results of two immune checkpoints (PDCD1, CTLA4) in 24 pancreatic cancer tissue samples. **(K–R)** Results of Spearmancorrelation analysis between the four genes (FYN, CASP4 and TOB1) and the two immune checkpoints (PDCD1, CTLA4).

Regarding the H&E staining of adjacent normal tissue and tumor tissue, pancreatic cancer tissue exhibited a higher infiltration of immune cells. (**Figures 10A–B**) Immunohistochemistry staining of pancreatic cancer tissues and adjacent normal tissues was performed using CD3/FOXP3 markers, revealing higher infiltration of T/NK cells and Treg cells in tumor tissues compared to adjacent normal tissues (**Figures 10C–D**). Further CD4/FOXP3 immunofluorescence co-staining confirmed the higher levels of infiltration of Treg cells in pancreatic cancer tissue (**Figures 10E–F**). To validate our findings, we conducted PCR analysis to assess the relative expression of the four identified genes in a cohort of 24 patients with available survival data (**Figures 11A–D**). Subsequently, survival analysis was conducted using the Kaplan-Meier (K-M) method. The results confirmed our previous conclusions, demonstrating that CASP4 (P = 0.0034), CLEC2B (P = 0.0011), and TOB1 (P = 0.0013) exhibited a significantly negative correlation with patient prognosis. Conversely, FYN (P = 0.00062) was identified as a protective factor for prognosis (**Figures 11E–H**). To further corroborate our immunoassay results, we investigated the correlation between two representative immune checkpoints and the four identified genes (**Figures 11I–J**). Correlation analysis revealed that CASP4 exhibited a moderate correlation with PDCD1 (R = 0.47, P = 0.0022) and a strong correlation with CTLA4 (R = 0.72, P = 0.00012). CLEC2B demonstrated a strong correlation with PDCD1 (R = 0.56, P = 0.0043) and CTLA4 (R = 0.7, P = 0.00021). Conversely, FYN showed no significant association with PDCD1 (R = 0.14, P = 0.51) or CTLA4 (R = 0.16, P = 0.45). Notably, TOB1 displayed a strong correlation with PDCD1 (R = 0.72, P < 0.001) and CTLA4 (R = 0.57, P = 0.0041) (**Figures 11K–R**).

## 4 Discussion

Approximately thirty years ago, Sakaguchi et al. reported the discovery of a unique cluster of CD4+ CD25+ T cells with immunosuppressive functions, which they named regulatory T cells (Treg) (70). Subsequent studies have confirmed that Treg cells represent a diverse population of T cells, predominantly expressing CD4 molecules on their cell surface. Despite their relatively small proportion, accounting for about 1% of developing CD4 single-positive thymocytes and 10% to 15% of CD4+ T cells in secondary lymphoid organs, Treg cells play crucial roles (71, 72). Treg cells can be classified into two subsets based on their developmental sites. Natural Treg cells (nTreg) are generated in the thymus, while induced Treg cells (iTreg) are derived from peripheral naïve T cells in response to TCR stimulation with retinoic acid or TGF-β (73–75). Treg cells possess potent immunosuppressive capabilities and exert specific and nonspecific regulatory effects on the immune system. They achieve this by inhibiting dendritic cell function and maturation, secreting anti-inflammatory cytokines, and suppressing the induction and proliferation of antigen-specific effector T cells (Teff) (44-46). Treg cells prevent immune-mediated attacks on self-tissues and cells, promoting immune tolerance to autologous components and maintaining immune homeostasis. Over the years, research has highlighted the significant role of Treg cells in various pathological processes, including autoimmune diseases and organ transplant rejection. Moreover, a growing understanding of the tumor microenvironment has revealed the importance of Treg cells in cancer, elucidating certain mechanisms through which they contribute to tumor progression (76–78).

The high expression of immune checkpoints on the surface of Treg cells and antigen-presenting cells (APCs) directly suppresses APC activity and hinders their ability to activate conventional effector T cells through interactions with Treg cells (79, 80). Additionally, the overexpression of CD39 on Treg cells facilitates the conversion of adenosine triphosphate (ATP) to adenosine. Adenosine, in turn, binds to A2A receptors (A2AR) and/or A2B receptors (A2BR) expressed on dendritic cells, effector T cells, and natural killer (NK) cells, resulting in immunosuppression (81, 82). Furthermore, studies have demonstrated that Treg cells directly induce cytotoxicity in effector T and NK cell populations by secreting perforin and granzyme (83). Given the prominent role of Treg cells in promoting immune tolerance within the tumor microenvironment, researchers have developed various therapeutic approaches targeting Treg cells, such as CTLA-4 blockade (84), CD25 modulation (85), and interventions involving receptor superfamilies including tumor necrosis factor receptor (TNFR), immunoglobulin, immune checkpoint receptor, and G protein-coupled receptor (GPCR) superfamily proteins (86, 87).

Alterations in metabolism and cytokines in the microenvironment of lesions of disease greatly influence disease progression (88, 89). In recent years, with the advancement of immune microenvironment studies, it has been increasingly recognized that certain cells, previously considered “accomplices” in tumor progression, may also exhibit inhibitory effects on tumor growth (90, 91). This phenomenon is also observed in Treg cells. A study conducted by Yaqing Zhang et al. (92) investigated this aspect in a mouse model of pancreatic cancer. Upon depletion of Treg cells, mice exhibited a robust inflammatory response, with the inflammation in the pancreas synergistically promoting pancreatic cancer development driven by the carcinogenic Kras mutation. Furthermore, Treg cell depletion also influenced the number and function of other cells. For instance, immunosuppressive bone marrow cells significantly increased, while tumor-associated macrophages demonstrated heightened immunosuppressive capacity. Similar findings have been supported by other researchers (93, 94). Therefore, the clinical value of Treg cell depletion therapy and the delineation of the tumor suppressor/tumor promoter role of Treg cells in clinical treatment warrant further investigation (53). It is essential to address these questions to gain a comprehensive understanding of the therapeutic potential and limitations associated with targeting Treg cells in cancer treatment.

In our study, Treg cells played an important role in the tumor microenvironment of pancreatic cancer. In the T cell cluster, the Treg cell was more mature, but even those identified as Treg cell was still in different branches on the differentiation trajectory. This seemed to indicate that although the infiltrating Treg cell in the microenvironment of pancreatic cancer has the main function of inhibiting tumor immunity and assisting tumor escape after differentiation and maturation, there might be some differences in the way of achieving their main functions or secondary functions. In addition, the communication between cells in the pancreatic cancer microenvironment was extremely close, and a variety of traditional tumor signaling pathways were significantly activated. After our statistics, among the 38 abnormal activated signaling pathways identified by cell communication analysis, Treg cells were involved in 27 pathways (CD46, TNF, ITGB2, and other signaling pathways), of which 15 pathways bear the identity of important participants (in addition to the five pathways analyzed above, there were GRN, APP, GALECTIN, IL-16, and other signaling pathways). Only 11 pathways were not active (including NOTCH, CCL, EPHA, etc.). The number of Treg cells infiltrated in different tumor samples was not constant, and there was no significant difference in the immune infiltration analysis of large samples in the GEO cohort. Nevertheless, Treg cell still through the functional transformation in the infiltration of pancreatic cancer has become an important accomplice, that making it difficult for anti-tumor immunity to kill tumor cells effectively.

Moreover, in our study, we investigated the potential mechanisms underlying Treg cell heterogeneity using single-cell sequencing data. The four Treg cell marker genes identified in this study were found to be involved in the development and differentiation of Treg cells, as confirmed by pseudo-timing analysis. Furthermore, through our subsequent classification and hdWGCNA analysis, we identified the HIF-1 signaling pathway as a critical pathway associated with Treg cell heterogeneity. Hypoxia is a characteristic feature of the tumor microenvironment. As the tumor grows, it becomes distanced from blood vessels, and the dense interstitial cell population within the tumor microenvironment contributes to a hypoxic milieu (95). To adapt and thrive in this hypoxic environment, tumor cells undergo metabolic reprogramming by activating the HIF-1 signaling pathway, which enhances their anaerobic metabolism capabilities (96). Further studies have demonstrated that HIF-1 signaling pathway activation is not limited to tumor cells but is also observed in other cell types. For instance, HIF-1α has been found to bind to FOXP3 and promote FOXP3 degradation, thereby inhibiting Treg cell differentiation (97). HIF-1α regulates T cell metabolism, including glycolysis, which in turn inhibits Treg cell development (98). Additionally, targeting HIF-2α to disrupt Treg cells may represent a potential approach to modulate the functional activity of Treg cells (99). Therefore, we propose that the four identified TRGs have significant potential to modulate the “double-edged sword” effect of Treg cells by influencing the HIF-1 signaling pathway. These findings highlight the importance of understanding the role of Treg cell heterogeneity and its regulation, particularly through the involvement of the HIF-1 signaling pathway.

During this investigation, we identified four TRGs that exhibited significant expression in Treg cells, directly correlating with Treg cell infiltration in pancreatic cancer and influencing disease progression. Let’s delve into the characteristics and roles of these genes. CASP4, an inflammatory caspase, plays a crucial role in the innate immune response by facilitating the fusion of phagosomes and lysosomes carrying pathogens. It inhibits intracellular pathogen replication and promotes the maturation and secretion of pro-inflammatory cytokines. Knockdown of CASP4 has been shown to impede cell migration, cell-matrix adhesion, and tissue invasion in epithelial cancer cell lines (100). Fyn, a member of the Src kinase family (SFK), is a non-receptor tyrosine kinase involved in signal transduction pathways in the nervous system, as well as T lymphocyte formation and activation. Numerous studies have revealed that Fyn overexpression directly promotes the proliferation and invasion of various tumor cell lines, potentially through the Ras/PI3K/Akt signaling pathway (101, 102). TOB1, an anti-proliferative protein from the Tob/BTG family, is often implicated in tumorigenesis and T cell activation. TOB1’s promotion of tumor cell lines may be attributed to its activation of classical tumor pathways such as WNT, JNK, and P38 (103–105). CLEC2B is predominantly expressed in human platelets/megakaryocytes and functions to activate platelets and facilitate coagulation. Studies have also suggested that CLEC2B activation in platelets plays a crucial role in development, inflammation, and cancer (106, 107). Although previous studies have shed light on the potential mechanisms underlying the expression changes of these four TRGs in promoting tumor cells, the focus has primarily been on their effects within tumor cell lines. The mechanisms by which altered expression of TRGs influences Treg cells and promotes tumor progression remain elusive. Moreover, research investigating the relationship between TRGs and pancreatic cancer is limited. Therefore, we believe that further investigation into the co-culture of Treg cells and pancreatic cancer cells is necessary to elucidate the associated mechanisms.

Prior research has employed bioinformatics methodologies to explore the correlation between immune cells and tumors. However, the methods utilized were outdated, and there was a lack of relevant investigations into the mechanisms underlying Treg involvement in regulating pancreatic cancer. In contrast to previous studies, this research incorporates state-of-the-art statistical analysis techniques and integrates sequencing data from various platforms. This not only facilitates a more comprehensive and rigorous analysis of key genes in Treg cells but also introduces novel approaches to transcriptomic studies of the interaction between tumors and immune cells. However, there were certain limitations to our research. Although our conclusions were primarily based on bioinformatics analysis, additional experimental validation was necessary to support our findings. While we conducted some *in vitro* validation, such as immunofluorescence staining and PCR analysis, the limited availability of pancreatic cancer patient samples hindered us from obtaining a sufficiently large sample size. Nonetheless, the experimental results we obtained align closely with our bioinformatics analysis conclusions, providing substantial evidence. In addition, conducting cytological experiments or organoid experiments involving the co-culture of Treg cells with pancreatic ductal adenocarcinoma cells could potentially provide further insights and enhance the evidential basis for our research.

Moreover, our research provided valuable recommendations for clinical practice. Immunological checkpoints, which involve ligand-receptor interactions that either suppress or enhance immune responses, play a critical role in maintaining self-tolerance and regulating the duration and intensity of immune reactions to minimize tissue damage. It has been increasingly recognized that tumor types often exhibit the expression of inhibitory or stimulatory immune checkpoint molecules (108–110). Consequently, the expression of immune checkpoints can serve as a descriptor for tumor behavior patterns and responsiveness to targeted therapies. In our study, we observed that patients in the high-risk group exhibited a high expression of numerous checkpoints, as determined by the stratification of patients based on TRG expression levels. To enhance treatment efficacy, a potential approach was to consider dual-targeted therapy that simultaneously targeted TRGs and specific immune checkpoints based on their expression patterns. Additionally, we predicted the potential therapeutic sensitivity of traditional chemotherapy drugs for both patient groups. We also identified potential therapeutic agents that could disrupt the immunosuppressive effects mediated by Treg cells within the pancreatic cancer microenvironment, thereby enhancing the clinical effectiveness of pancreatic cancer treatments. These findings offered practical implications for improving treatment outcomes in pancreatic cancer and suggested avenues for personalized therapeutic interventions targeting both TRGs and immune checkpoints, as well as identifying potential drugs to counteract Treg cell-mediated immunosuppression.

## 5 Conclusion

In conclusion, our study provided evidence of Treg cell infiltration within the microenvironment of pancreatic cancer. We analyzed the differentiation status of Treg cells within the T/NK cell cluster and investigated their communication pathways with other cells. Moreover, we identified four Treg cell-related genes that significantly contributed to the development and progression of pancreatic cancer. Importantly, we examined the clinical relevance of these genes based on their expression patterns. Overall, our findings shed light on the role of Treg cells in pancreatic cancer and elucidated the significance of the identified Treg cell-related genes. This research added to our understanding of the molecular mechanisms underlying pancreatic cancer and paved the way for potential therapeutic interventions targeting Treg cells in this disease.

## Data availability statement

The original contributions presented in the study are included in the article/**Supplementary Material**. Further inquiries can be directed to the corresponding authors.

## Ethics statement

The studies involving human participants were reviewed and approved by Ethics Committee of the First Affiliated Hospital of Soochow University. The patients/participants provided their written informed consent to participate in this study.

## Author contributions

CFX, WXZ, and QL conceived the study. WX, DXZ, and QW drafted the manuscript. WX and MZ performed the literature search and collected the data. WX and DXZ analyzed and visualized the data. WX, WJZ and DXZ completed all experiments. CFX, WXZ, WJZ and QL helped with the final revision of this manuscript. All authors reviewed and approved the final manuscript.

## Supplementary material

Supplementary Figure 1 Flow chart

Supplementary Figure 2 Expression of selected marker genes for cell type annotation in each subpopulation

Supplementary Figure 3 Performance and ligand-receptor display of Treg cells in CD99 pathway. **(A, B)** Total communication intensity of each cell in the CD99 pathway. **(C)** The heatmap quantitatively showed the total communication intensity of each cell in the CD99 pathway. **(D)** All ligand-receptor contribution values in the CD99 pathway. **(E)** Each cell was based on CD99-CD99 ligand-receptor exchange intensity. **(F)** Visualization of the communication intensity of individual cells as signal transmitters and receivers in the CD99 pathway. **(G)** The expression of each receptor in the CD99 pathway between cells. **(H)** The heatmap of the intensity of communication between individual cells in the CD99 pathway as signal senders and receivers in the CD99 pathway.

Supplementary Figure 4 Performance and ligand-receptor display of Treg cells in FN1 pathway. **(A, B)** Total communication intensity of each cell in the FN1 pathway. **(C)** The heatmap quantitatively showed the total communication intensity of each cell in the FN1 pathway. **(D)** All ligand-receptor contribution values in the FN1 pathway. **(E)** Each cell was based on FN1-CD41 ligand-receptor exchange intensity. **(F)** Visualization of the communication intensity of individual cells as signal transmitters and receivers in the FN1 pathway. **(G)** The expression of each receptor in the FN1 pathway between cells. **(H)** The heatmap of the intensity of communication between individual cells in the FN1 pathway as signal senders and receivers in the FN1 pathway.

Supplementary Figure 5 Performance and ligand-receptor display of Treg cells in MHC-II pathway. **(A, B)** Total communication intensity of each cell in the MHC-II pathway. **(C)** The heatmap quantitatively showed the total communication intensity of each cell in the MHC-II pathway. **(D)** All ligand-receptor contribution values in the MHC-II pathway. **(E)** Each cell was based on HLA-DRA-CD4 ligand-receptor exchange intensity. **(F)** Visualization of the communication intensity of individual cells as signal transmitters and receivers in the MHC-II pathway. **(G)** The expression of each receptor in the MHC-II pathway between cells. **(H)** The heatmap of the intensity of communication between individual cells in the MHC-II pathway as signal senders and receivers in the MHC-II pathway.

Supplementary Figure 6 Performance and ligand-receptor display of Treg cells in MIF pathway**. (A, B)** Total communication intensity of each cell in the MIF pathway. **(C)** The heatmap quantitatively showed the total communication intensity of each cell in the MIF pathway. **(D)** All ligand-receptor contribution values in the MIF pathway. **(E)** Each cell was based on MIF-(CD47+CD44) ligand-receptor exchange intensity. **(F)** Visualization of the communication intensity of individual cells as signal transmitters and receivers in the MIF pathway. **(G)** The expression of each receptor in the MIF pathway between cells. **(H)** The heatmap of the intensity of communication between individual cells in the MIF pathway as signal senders and receivers in the MIF pathway.

Supplementary Figure 7 Performance and ligand-receptor display of Treg cells in MPZ pathway. **(A, B)** Total communication intensity of each cell in the MPZ pathway. **(C)** The heatmap quantitatively showed the total communication intensity of each cell in the MPZ pathway. **(D)** All ligand-receptor contribution values in the MPZ pathway. **(E)** Each cell was based on MPZL1- MPZL1 ligand-receptor exchange intensity. **(F)** Visualization of the communication intensity of individual cells as signal transmitters and receivers in the MPZ pathway. **(G)** The expression of each receptor in the MPZ pathway between cells. **(H)** The heatmap of the intensity of communication between individual cells in the MPZ pathway as signal senders and receivers in the MPZ pathway.

Supplementary Table 1 Primer sequences for PCR detection of genes
