# Supplementary figures and images for: Unveiling the role of regulatory T cells in the tumor microenvironment of pancreatic cancer through single-cell transcriptomics and in vitro experiments
Source: Front Immunol. 2023 Sep 11;14:1242909. doi: 10.3389/fimmu.2023.1242909 (PMC10518406; doi:10.3389/fimmu.2023.1242909)

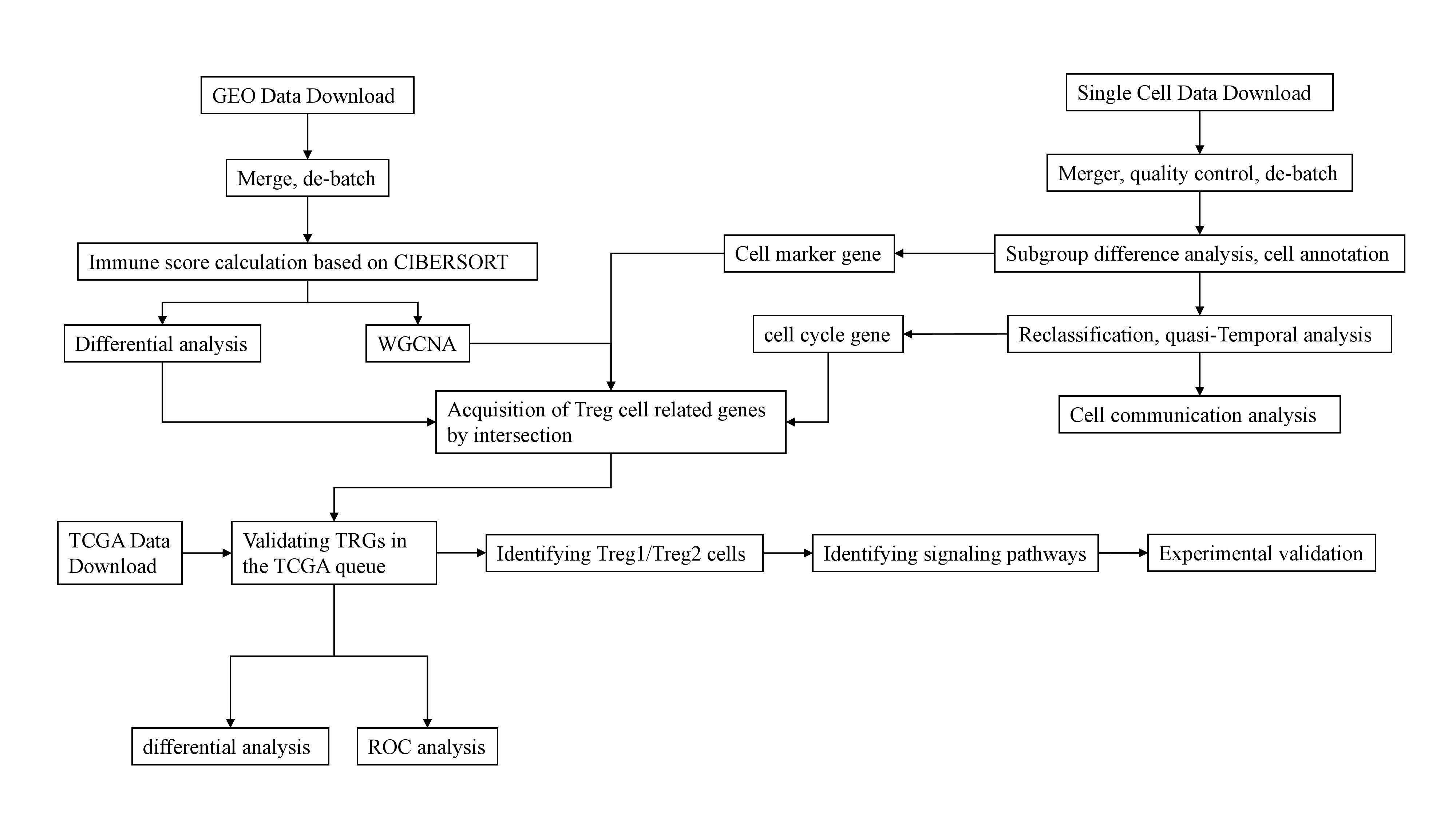

Supplement: Supplementary Figure 1 — Flow chart [file Image_1.tif]
