# Supplementary material for: Unveiling the role of regulatory T cells in the tumor microenvironment of pancreatic cancer through single-cell transcriptomics and in vitro experiments
Source: Front Immunol. 2023 Sep 11;14:1242909. doi: 10.3389/fimmu.2023.1242909 (PMC10518406; doi:10.3389/fimmu.2023.1242909)

Acinar


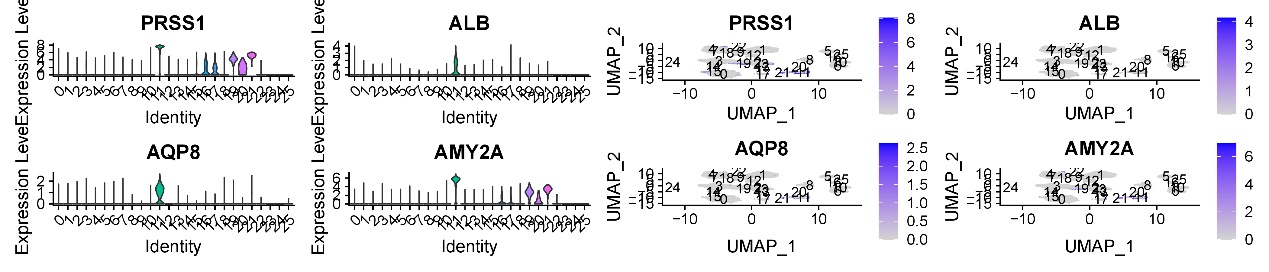


B cell


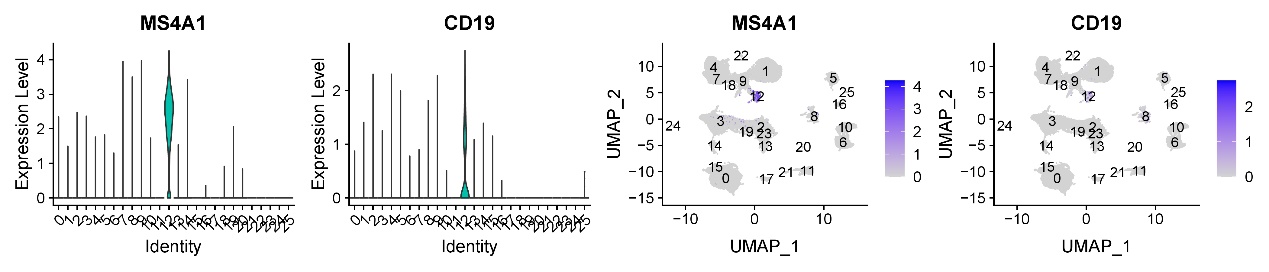


DC


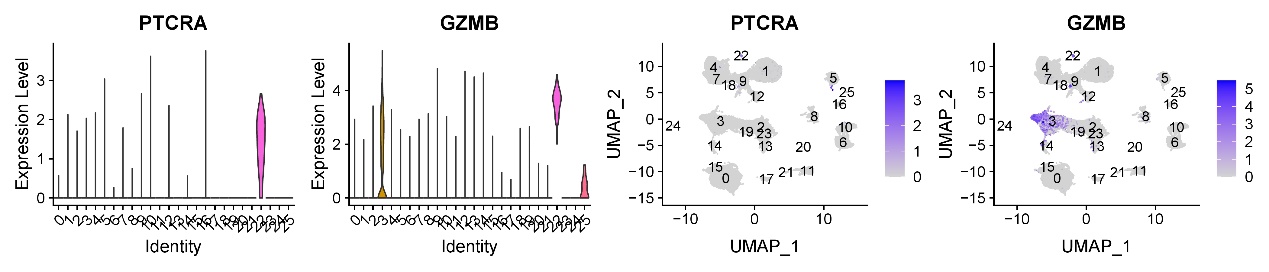


Duct


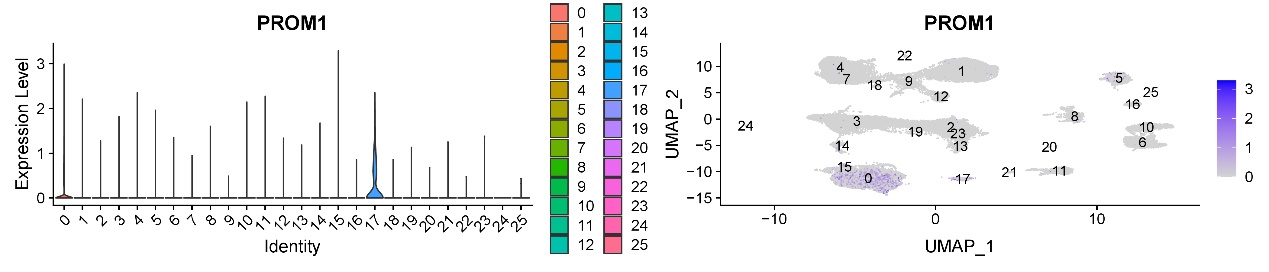


Endocrine


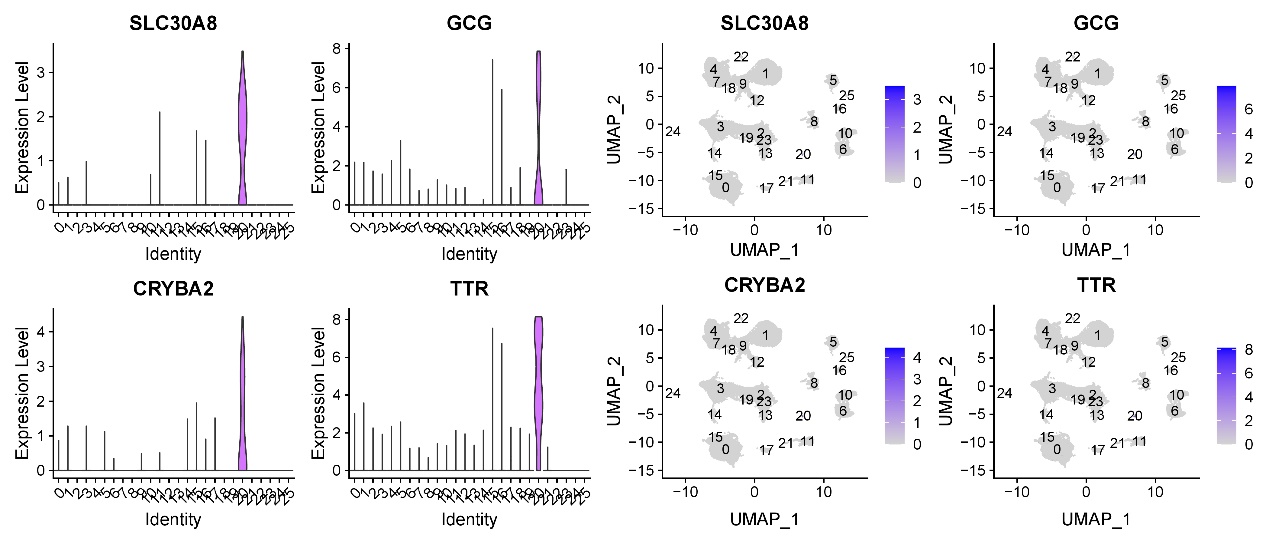


Endothelial


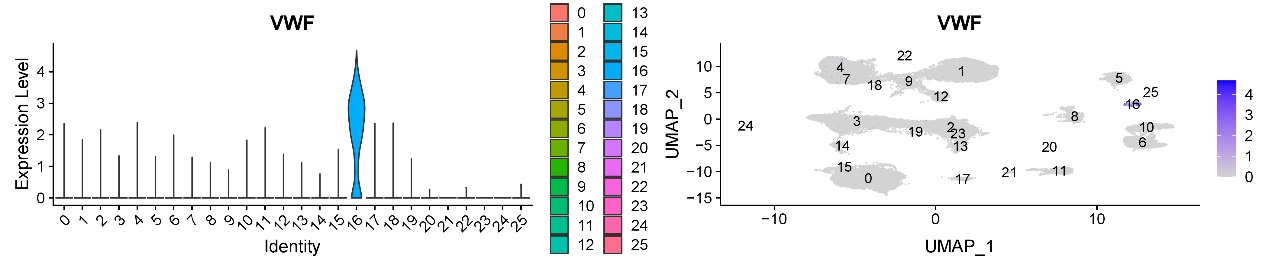


Epithelial


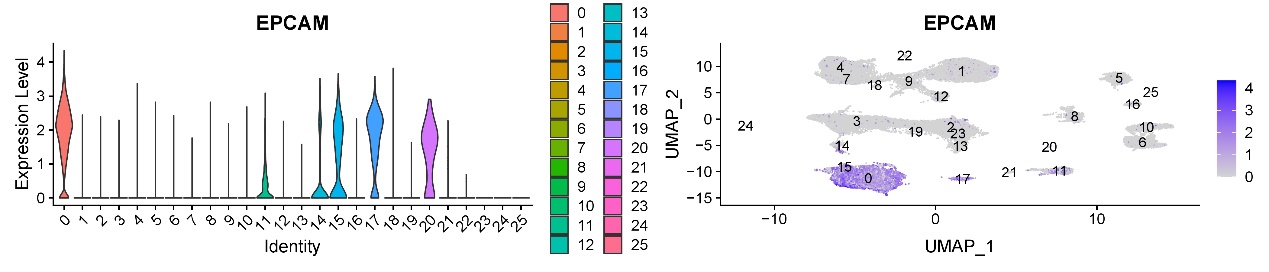


Fibroblast


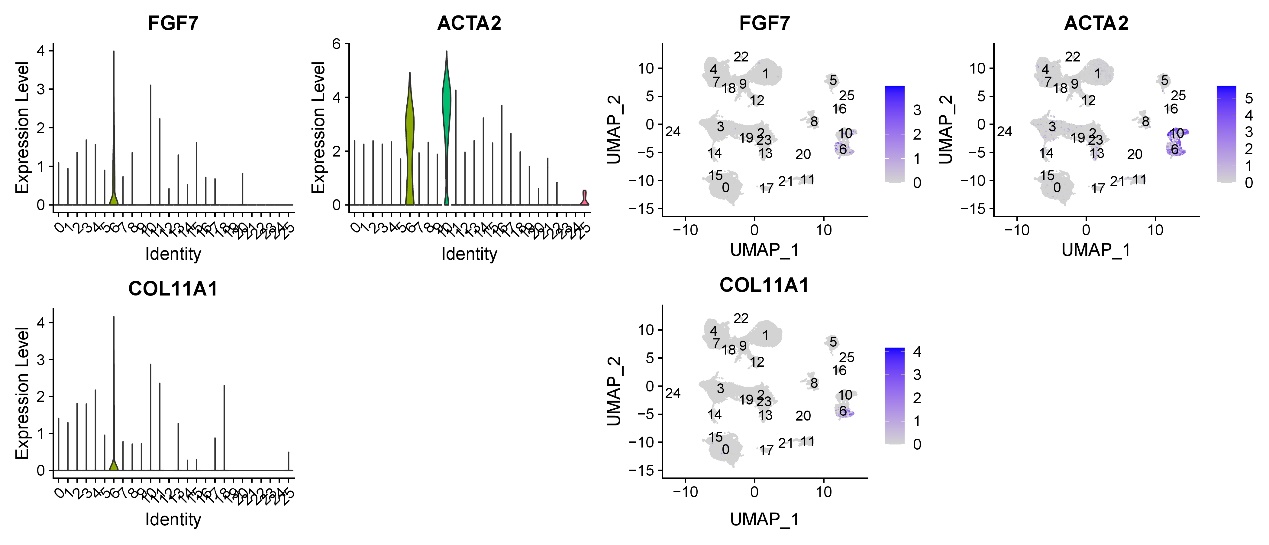


imm_stro_cancer


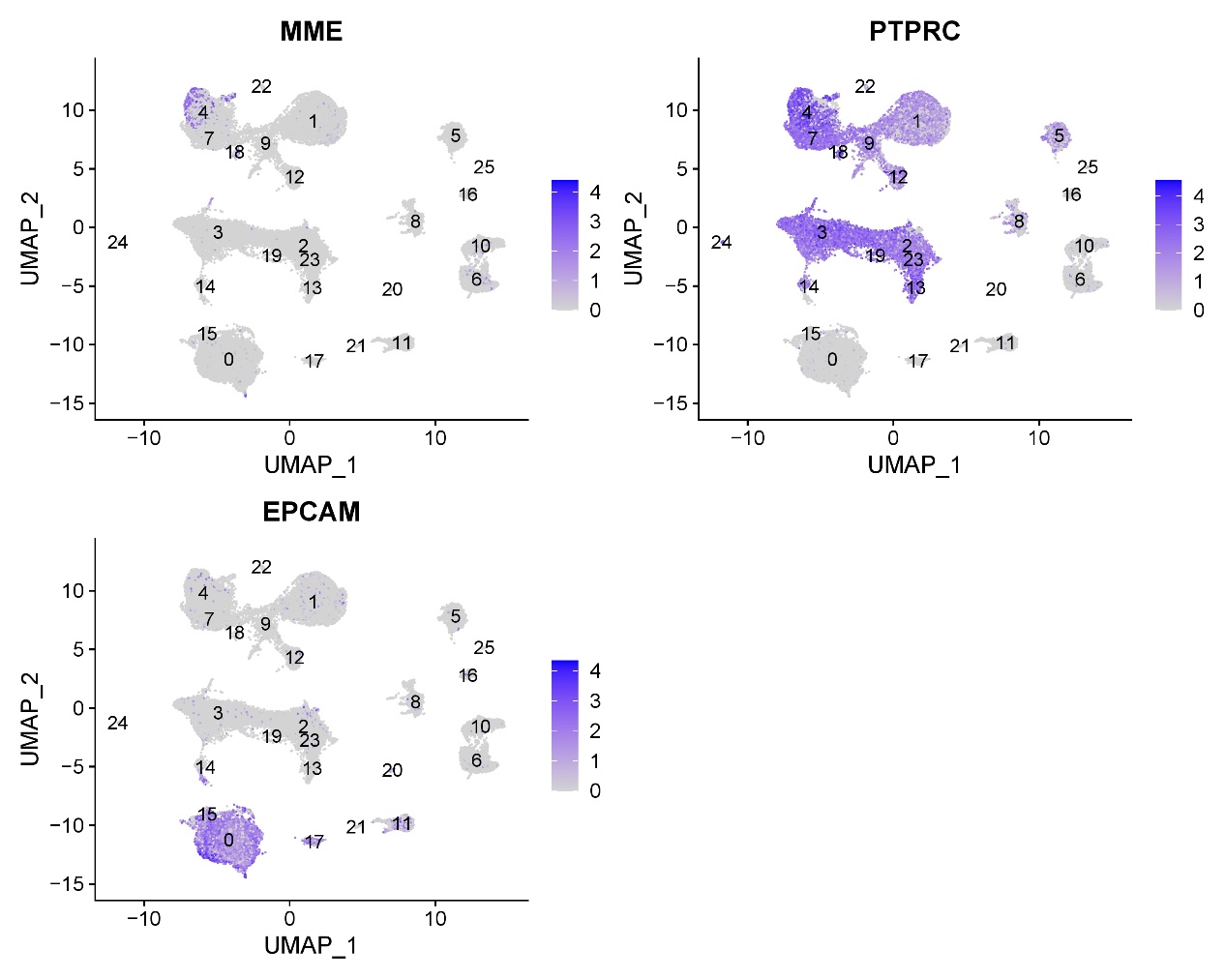


Macrophage


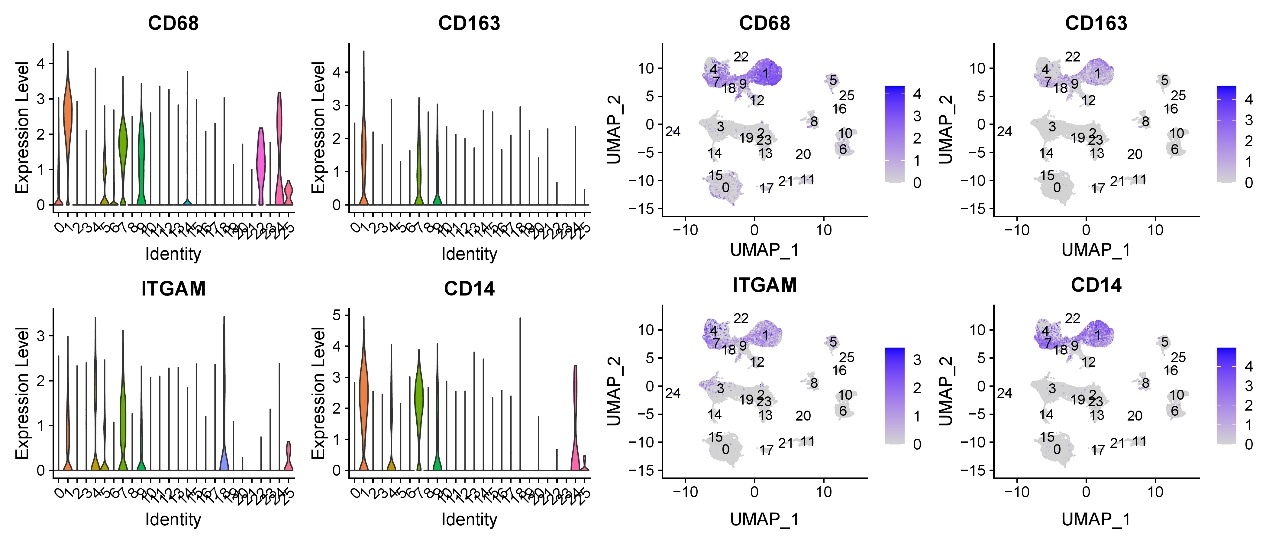


Mast


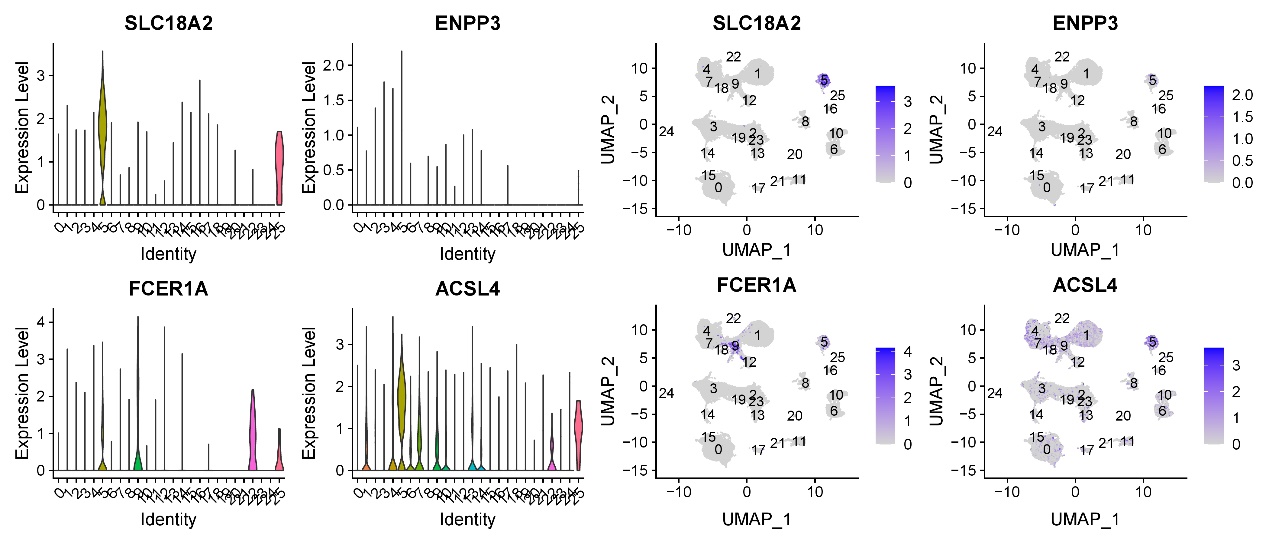


Neutrophil


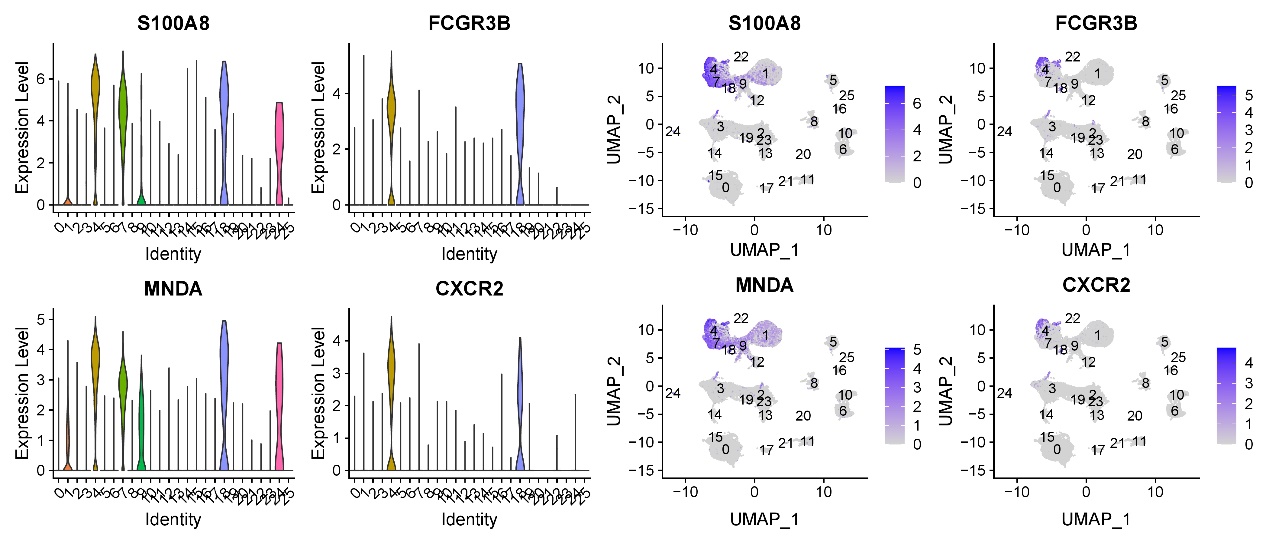


plasma


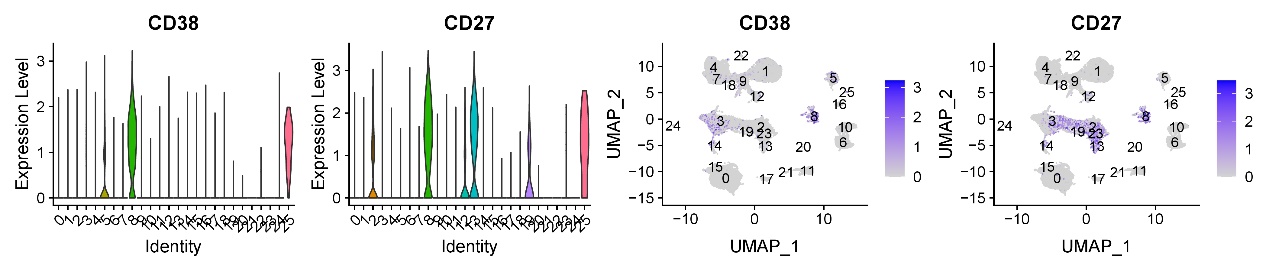


Progenitor


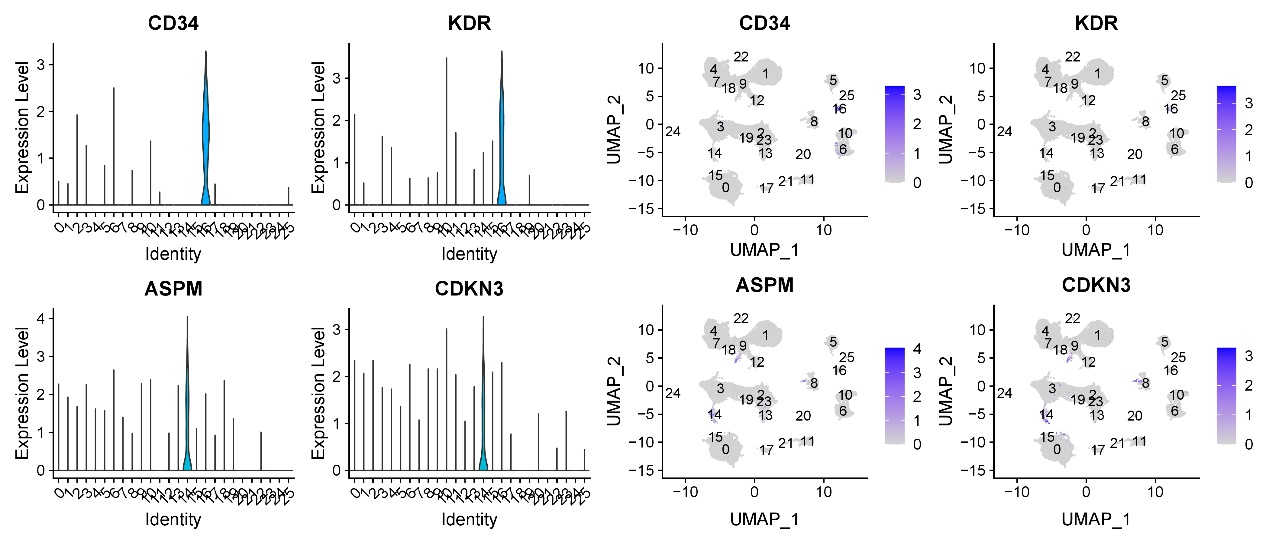


T_NK


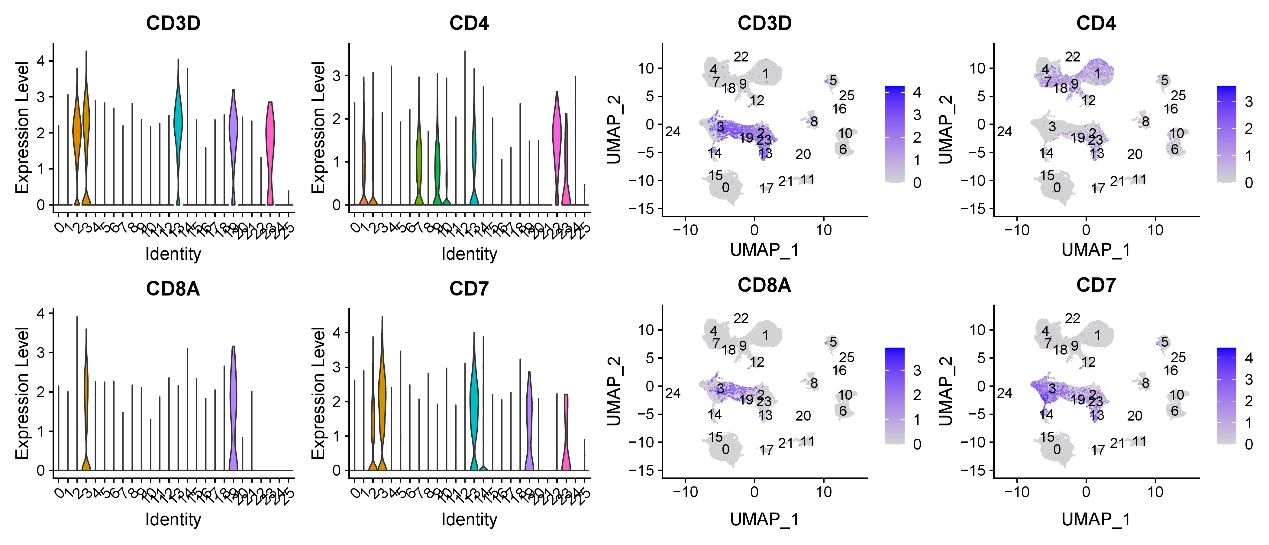

Supplement: Supplementary Figure 2 — Expression of selected marker genes for cell type annotation in each subpopulation [file DataSheet_1.docx]
