# Supplementary material for: Unveiling the role of regulatory T cells in the tumor microenvironment of pancreatic cancer through single-cell transcriptomics and in vitro experiments
Source: Front Immunol. 2023 Sep 11;14:1242909. doi: 10.3389/fimmu.2023.1242909 (PMC10518406; doi:10.3389/fimmu.2023.1242909)

**Supplementary figure3**


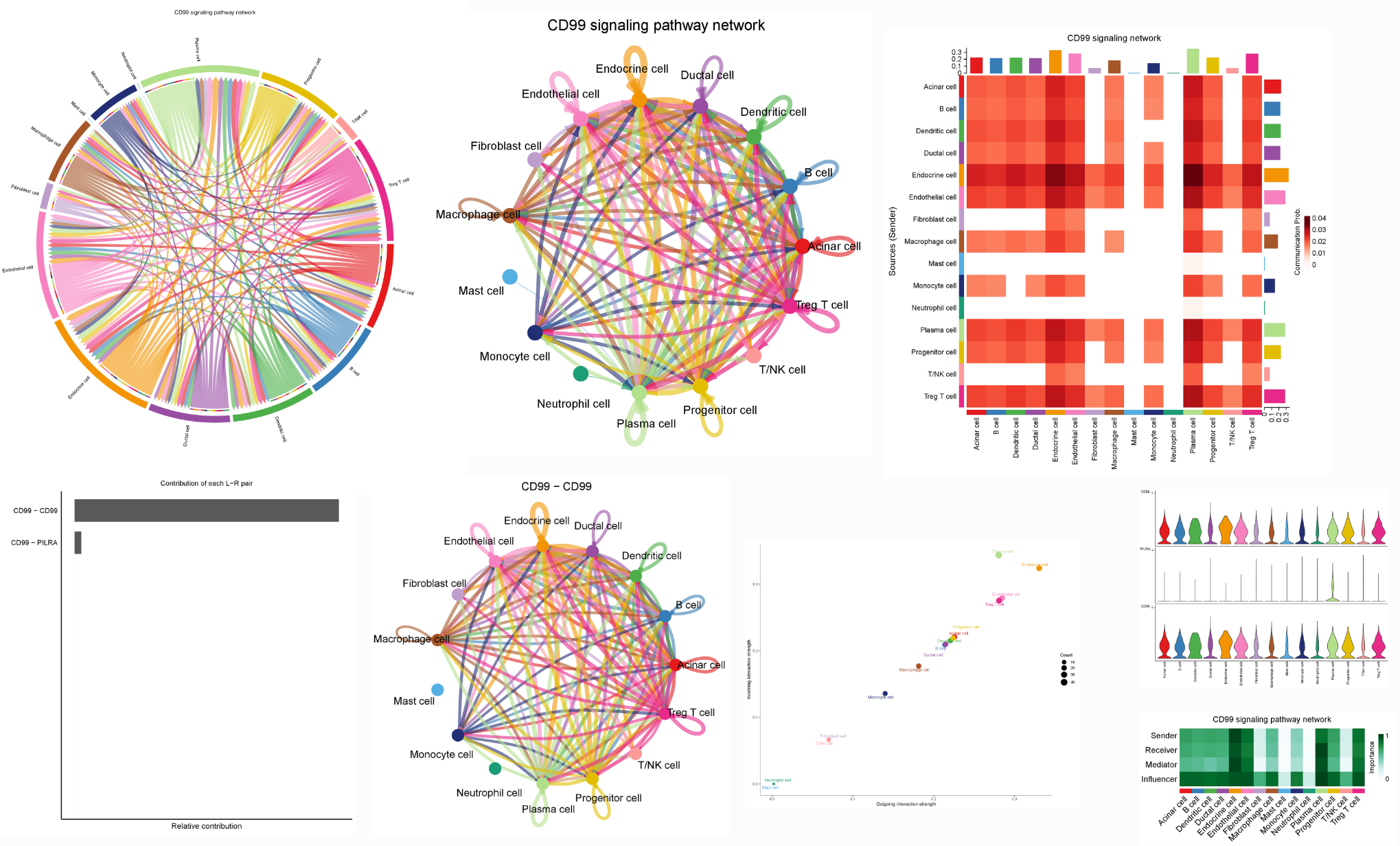


**Supplementary figure4**


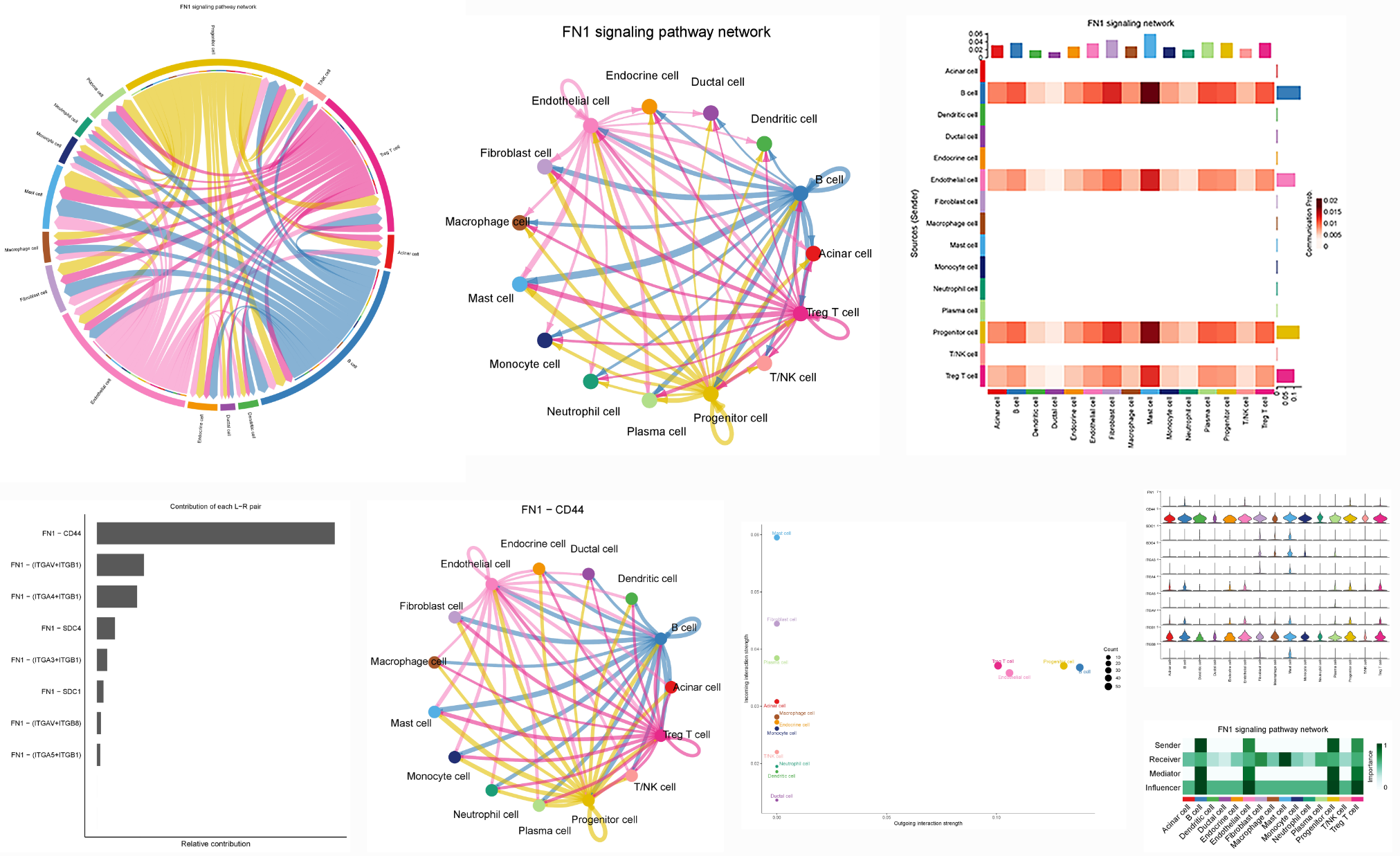


**Supplementary figure5**


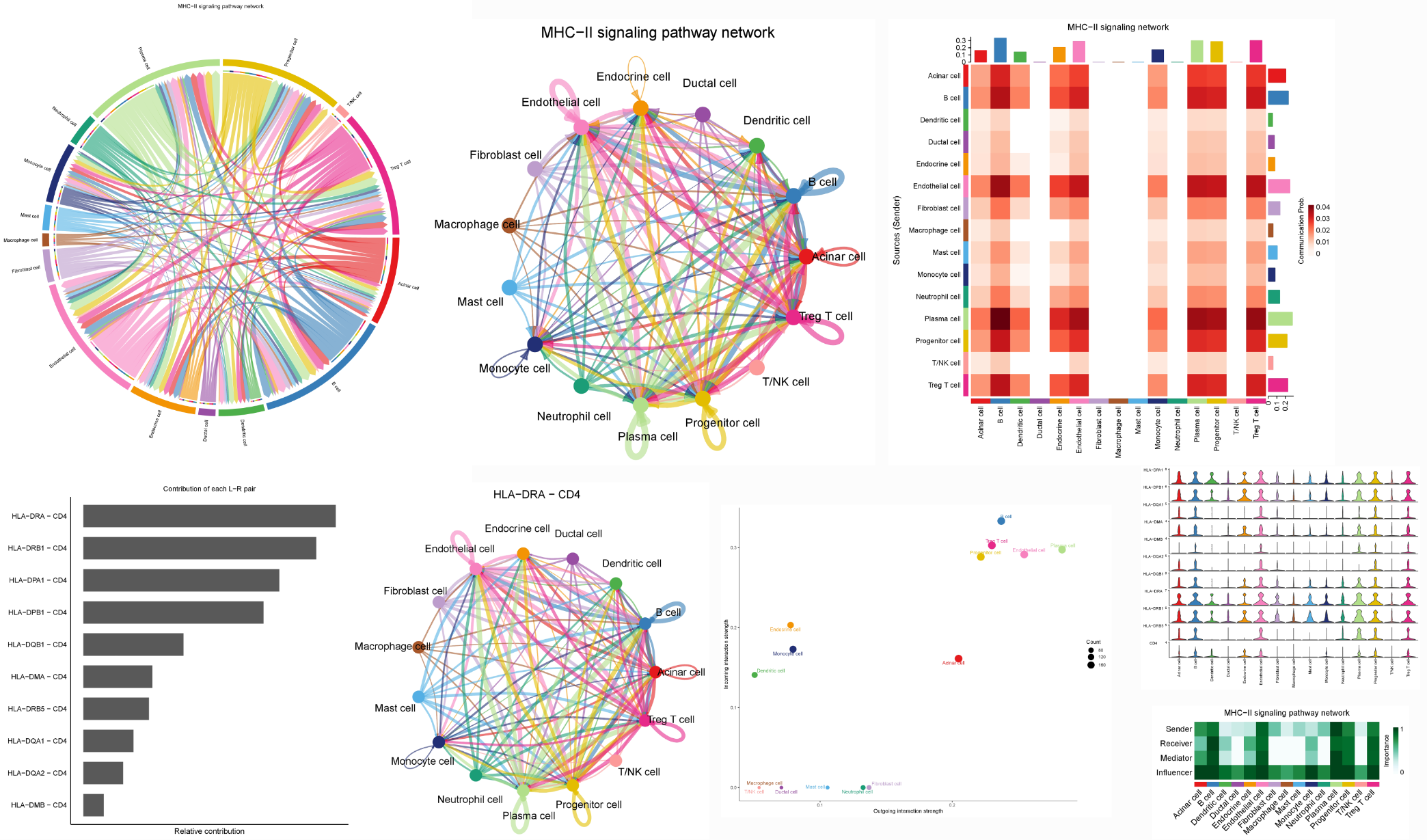


**Supplementary figure6**


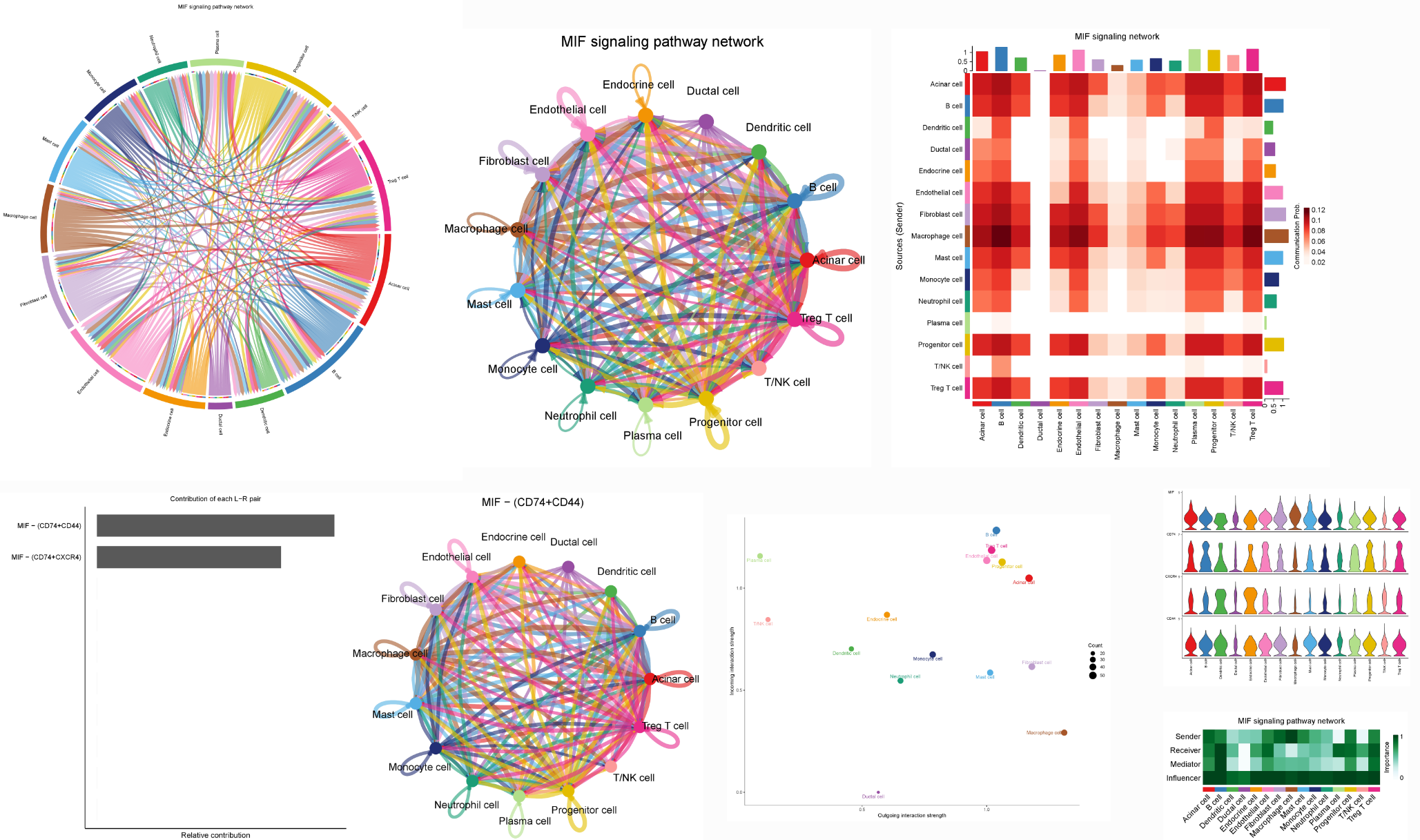


**Supplementary figure7**


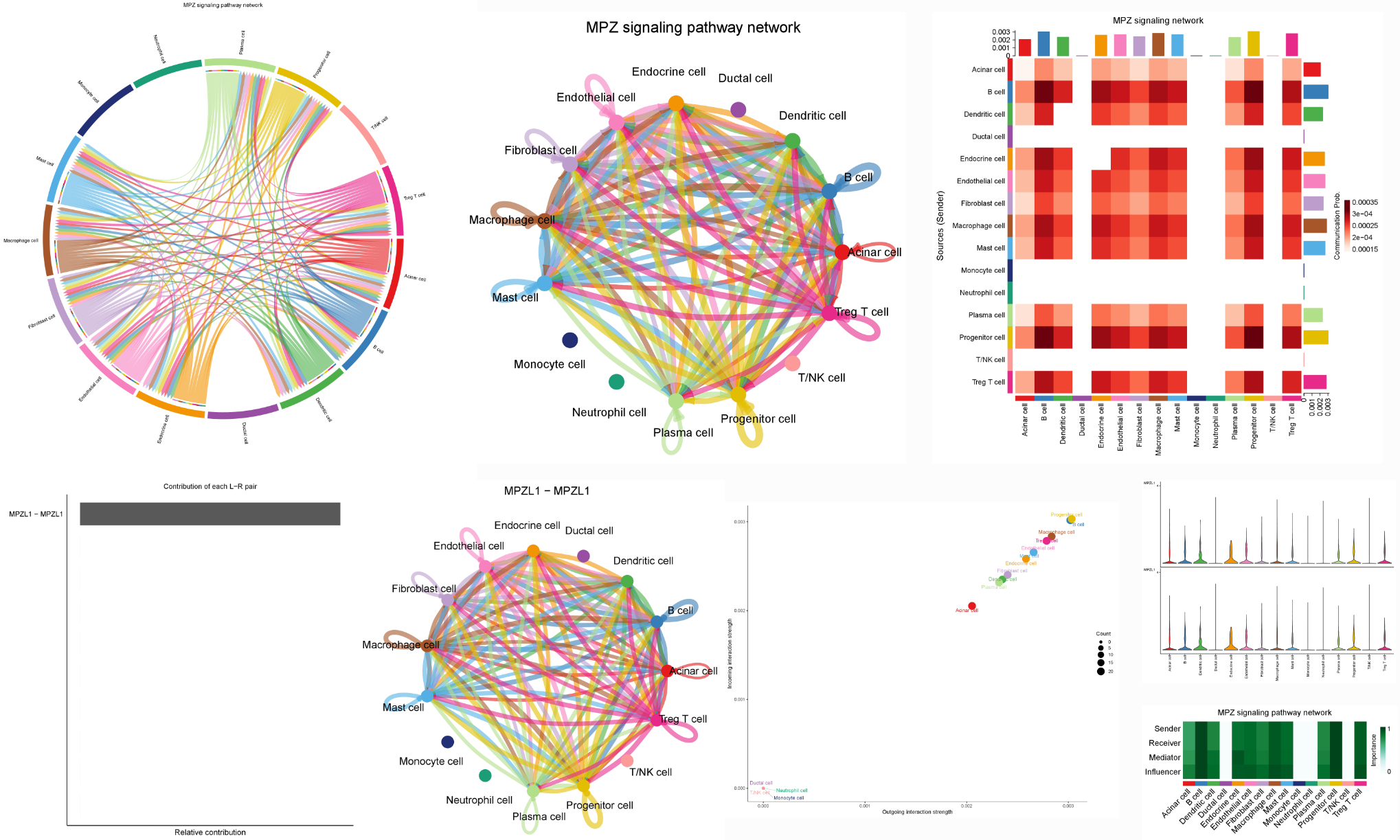

Supplement: Supplementary Figure 3 — Performance and ligand-receptor display of Treg cells in CD99 pathway. (A, B) Total communication intensity of each cell in the CD99 pathway. (C) The heatmap quantitatively showed the total communication intensity of each cell in the CD99 pathway. (D) All ligand-receptor contribution values in the CD99 pathway. (E) Each cell was based on CD99-CD99 ligand-receptor exchange intensity. (F) Visualization of the communication intensity of individual cells as signal transmitters and receivers in the CD99 pathway. (G) The expression of each receptor in the CD99 pathway between cells. (H) The heatmap of the intensity of communication between individual cells in the CD99 pathway as signal senders and receivers in the CD99 pathway. [file DataSheet_2.docx]
