# Supplementary material for: Unveiling the role of regulatory T cells in the tumor microenvironment of pancreatic cancer through single-cell transcriptomics and in vitro experiments
Source: Front Immunol. 2023 Sep 11;14:1242909. doi: 10.3389/fimmu.2023.1242909 (PMC10518406; doi:10.3389/fimmu.2023.1242909)
Supplement: Supplementary Table 1 — Primer sequences for PCR detection of genes [file Table_1.docx]

**CASP4**

F: AGGGCATTTGCTACCAGACC

R: GGCAGTTGCGGTTGTTGAAT

**FYN**

F: TCGTCTCATACGGGGACCTT

R: AGCTGTCGCTCAGCATCTTT

**TOB1**

F: GGTTGCACGTACTTCTCCCA

R: CCAAGCCAAGCCCATACAGA

**CLEC2B**

F: TTCCACTCAACATGCCGACC

R: CTCCCTCTCATGCCAAACGA

**PDCD1**

F: TTCCACATGAGCGTGGTCAG

R: TGGCTCCTATTGTCCCTCGT

**CTLA4**

F: AGGTGACTGAAGTCTGTGCG

R: CATGAGCTCCACCTTGCAGA
